# A large family of *Dscam* genes with tandemly arrayed 5′ cassettes in *Chelicerata*

**DOI:** 10.1038/ncomms11252

**Published:** 2016-04-15

**Authors:** Yuan Yue, Yijun Meng, Hongru Ma, Shouqing Hou, Guozheng Cao, Weiling Hong, Yang Shi, Pengjuan Guo, Baoping Liu, Feng Shi, Yun Yang, Yongfeng Jin

**Affiliations:** 1 Institute of Biochemistry, Innovation Center for Signaling Network, College of Life Sciences, Zhejiang University, Hangzhou, Zhejiang ZJ310058, China; 2 College of Life and Environmental Sciences; Hangzhou Normal University, Hangzhou, Zhejiang ZJ310036, China

## Abstract

*Drosophila* Dscam1 (Down Syndrome Cell Adhesion Molecules) and vertebrate clustered protocadherins (Pcdhs) are two classic examples of the extraordinary isoform diversity from a single genomic locus. *Dscam1* encodes 38,016 distinct isoforms via mutually exclusive splicing in *D. melanogaster*, while the vertebrate clustered *Pcdh*s utilize alternative promoters to generate isoform diversity. Here we reveal a shortened *Dscam* gene family with tandemly arrayed 5′ cassettes in *Chelicerata*. These cassette repeats generally comprise two or four exons, corresponding to variable Immunoglobulin 7 (Ig7) or Ig7–8 domains of *Drosophila* Dscam1. Furthermore, extraordinary isoform diversity has been generated through a combination of alternating promoter and alternative splicing. These *sDscams* have a high sequence similarity with *Drosophila Dscam1*, and share striking organizational resemblance to the 5′ variable regions of vertebrate clustered *Pcdh*s. Hence, our findings have important implications for understanding the functional similarities between *Drosophila Dscam1* and vertebrate *Pcdh*s, and may provide further mechanistic insights into the regulation of isoform diversity.

## Abstract (web summary)

*Drosophila Dscam1* and vertebrate clustered protocadherins (*Pcdh*) are known for their extraordinary isoform diversity. Here authors identify a shortened *Dscam* gene family in *Chelicerata*, which displays homology to *Drosophila Dscam1*, and employs splicing patterns similar to that of vertebrate *Pcdhs*.

## Graphical abstract

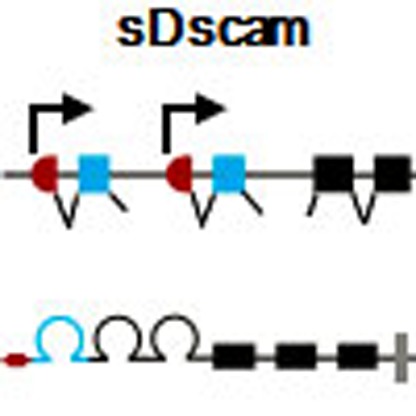

Alternative transcription and alternative splicing are two major means to expand the transcriptomic and proteomic repertoire from a single gene^1^,^2^. *Drosophila Dscam1* (Down Syndrome Cell Adhesion Molecules) and vertebrate clustered protocadherins (Pcdhs) are two classic examples of the extraordinary protein isoform diversity that can arise from a single complex genomic locus in two phyla^3^,^4^. *Dscam1* gene encodes 38,016 distinct isoforms via mutually exclusive alternative splicing of 4 arrays of tandem duplicated exons in *D. melanogaster*^3^. These Dscam1 isoforms are expressed stochastically and combinatorially, and exhibit isoform-specific homophilic binding^5^,^6^,^7^,^8^,^9^,^10^. These properties provide the molecular basis of *Drosophila* Dscam1 as a key molecule for self-avoidance, and genetic studies have indicated that thousands of Dscam1 isoforms are required for neuronal wiring and self-avoidance^8^,^9^,^10^,^11^,^12^,^13^,^14^. In contrast to insect *Dscam1*, vertebrate *Dscam* genes do not generate extraordinary protein diversity^15^.

However, another set of genes, the clustered *Pcdh*s, might perform the analogous function in vertebrates^16^,^17^,^18^. *Pcdh*s are the largest subgroup of the cadherin superfamily of cell adhesion proteins and are abundantly expressed in the central nervous system. In the human, 52 Pcdh proteins are encoded by 3 tightly linked gene clusters called *Pcdhα*, *Pcdhβ* and *Pcdhγ*, which are organized in a tandem array and on a single chromosome^4^. In these genes, each variable exon is preceded by a promoter, and Pcdh diversity is produced via differential promoter choice and *cis*-alternative splicing^19^,^20^. The *Pcdh* gene cluster encodes a large repertoire of cell surface recognition proteins, which can engage in specific homophilic interactions^21^. Functional experiments show that deletion of the mouse *Pcdhγ* gene cluster could cause defective dendritic self-avoidance in retinal starburst amacrine cells or in Purkinje cells^22^. This observation suggests that clustered *Pcdh*s, similar to *Drosophila Dscam1*, may also mediate neurite self-avoidance by specifying single-cell identity^21^,^22^,^23^,^24^. Conversely, such vertebrate clustered *Pcdh* genes have not been identified in *Drosophila*^16^.

Given the striking molecular parallels between and complementary phylogenetic distribution of Dscam diversity in *Drosophila* and the clustered *Pcdh* diversity in vertebrates, it is attractive to speculate that they may have similar roles. These two phyla appear to have evolved a common molecular strategy for self-avoidance by recruiting different molecules^18^. Nevertheless, since there is a big evolutionary gap between insects and vertebrates, who shared a common ancestor more than 500 million years ago, how the evolutionary transitions and complementarities occurred remains unclear. Moreover, *Drosophila Dscam1* generally generates tens of thousands of isoforms, while only 58 isoforms exist for clustered *Pcdh* genes in mice. This discrepancy in isoform diversity by at least 2 orders of magnitude is unlikely to be explained by the much higher common isoform tolerance for *Pcdh*s than is assumed for *Dscam1* (ref. 18).

In this study, we identified a novel Dscam gene family (*sDscam*) in *Chelicerata* that contained tandemly arrayed 5′ cassettes. The encoded proteins had a striking similarity to *Drosophila Dscam1*, but all lacked the canonical Immunoglobulin 1 (Ig1)–6, 10 and Fibronectin III (FNIII) 3–4, 6 domains present in classical DSCAM. The N-terminal domains of each *sDscam* protein are generally encoded by only one of a cluster of tandemly arrayed 5′ cassettes. These 5′ cassettes are generally comprised of two or four exons (*sDscamα* and *sDscamβ*), which correspond to variable Ig7 or Ig7–8 domains of *Drosophila* Dscam1. There was also high splicing complexity across variable 5′ clusters, which expanded the isoform diversity via a combination of alternative promoter and splicing activities. Thus, *Drosophila Dscam1* and *Chelicerata sDscam* represent examples of convergent evolution for isoform diversity. This genomic organization is remarkably similar to that of the clustered *Pcdh*s in vertebrates. Hence, our findings have important implications to aid in our understanding of the functional similarities between two structurally unrelated families of *Drosophila Dscam* and vertebrate *Pcdh*s, and may provide further insights into the regulatory mechanisms governing the selection of tandemly arrayed 5′ variable regions.

## Results

### A novel shortened *Dscam* gene family in *Mesobuthus martensii*

**Figure 1:**
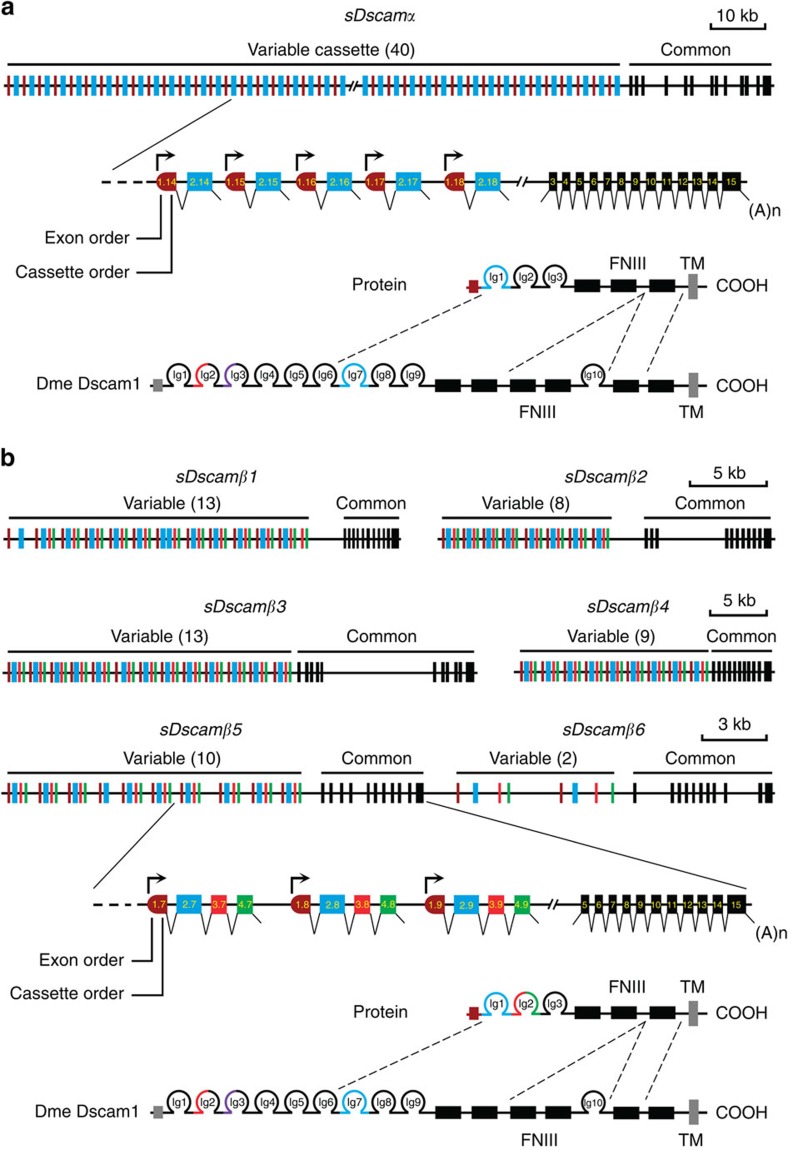
Organization of two novel *Dscam* gene subfamilies in *M. martensii*. (**a**) Organization of the *sDscamα* gene. The *sDscamα* gene is composed of multiple tandemly arrayed regions (indicated by the coloured boxes) and common region exons (indicated by the black boxes). ‘()' represents the number of tandem cassettes. The arrows indicate the transcription start sites. FNIII, fibronectin III domains; Ig, immunoglobulin domains. The N-terminal small boxes represent the leader peptides. The grey and black boxes represent the transmembrane (TM) and cytoplasmic domains, respectively. These cassettes are composed of two exons (indicated by the coloured boxes). Each variable cassette was transcribed by an alternative promoter followed by alternative splicing. The *sDscamα* variable cassette encoded the N-terminal Ig1 (blue), which corresponded to the variable Ig7 domain of *Drosophila Dscam1*. (**b**) Organization of the *sDscamβ* genes. This *sDscamβ* subfamily was composed of six members (*sDscamβ1–sDscamβ6*), 5′ tandem cassettes of which generally contained four exons. The variable cassette encoded the N-terminal Ig1+2 domains (coloured), which corresponded to the variable Ig7+8 domains of *Drosophila Dscam1*.

To trace the origins of duplicated exons of the *Dscam* genes in Arthropoda, the exons encoding the Ig7 orthologues of *Drosophila Dscam1* in the *M. martensii* genome were analysed. These Ig-coding exons were tandemly arrayed across the gene body, similar to *Drosophila Dscam1*. Nevertheless, RNA-seq analyses and sequencing of 5′ RACE (rapid-amplification of cDNA ends) products indicated that these transcripts shared no common upstream exons, and therefore, they might initiate immediately upstream of each variable exon (Fig. 1). Importantly, we believe this was located close to the transcription start sites for each variable exon, because a stop codon was generally located in the frame immediately upstream from the ATG initiation codon in each variable cassette (Supplementary Fig. 1). Last, computer-assisted and RNA-seq analyses revealed seven novel *Dscam* genes in *M. martensii*, which were characterized by tandemly arrayed 5′ cassettes (Fig. 1). Their encoding isoforms were similar to each other and to previously characterized *Drosophila Dscam1*, but all lacked the canonical Ig1–6,10 and FNIII 3–4, 6 domains present in classical DSCAM. We therefore designated these novel shortened *Dscam* genes as *sDscam*. Based on different units of tandemly arrayed 5′ cassettes, these *sDscams* could be subdivided into two closely related subfamilies, *sDscamα* and *sDscamβ* (Fig. 1a,b). The former (*sDscamα*) contained tandemly arrayed 5′ cassettes with 2 exons. This tandem cassette encoded a single Ig domain, which corresponded to the Ig7 of *Drosophila* Dscam1 (Fig. 1a). Genome-wide analyses revealed the presence of only one member of the *sDscamα* subfamily, which contained at least 40 tandem copies at the 5′ variable regions.

The tandemly arrayed 5′ cassette of another gene cluster subfamily (*sDscamβ*) generally contained 4 exons (Fig. 1b). These tandem cassettes encoded 2 Ig repeats, which corresponded to the Ig7–8 domains of *Drosophila* Dscam1. This is similar to Ig7–8 arrays in *Ixodes scapularis Dscam*, albeit without the annotation of the first exons^25^. We identified up to 6 members (*sDscamβ1–sDscamβ6*) of the *sDscamβ* subfamily, which contained 13, 8, 13, 9, 10 and 2 tandemly arrayed cassettes, respectively. In some cases, tandem cassettes could be made by the combination of different duplication units. Taken together, this unusual organization of the *sDscam* family potentiates the capacity to expand the transcript isoforms.

### *sDscam* 5′ clustered organization is conserved in *Chelicerata*

We examined whether this clustered organization of *sDscam* found in *M. martensii* was conserved at the 5′ variable regions throughout Arthropoda. This analysis was expanded to include the Araneae *Stegodyphus mimosarum*, 2 Ixodoidean species (*I. scapularis* and *Tetranychus urticae*) and Merostomatan *Limulus polyphemus.* Together, these organisms comprise some of the major taxonomic groups of the *Chelicerata* subphylum that last shared a common ancestor ∼420 million years ago^26^. We identified the clustered organization at the 5′ regions of *sDscam* in all species of the Arachnida class investigated, although the members of the tandemly arrayed 5′ cassettes differed among species (Supplementary Fig. 2). This led us to believe that the 5′ clustered organization of the *sDscam* family was evolutionarily conserved in Arachnida. Moreover, the sequence comparison revealed the 5′ clustered organization of the *sDscamα* and *sDscamβ* subfamilies in Merostomatan *L. polyphemus* (Supplementary Fig. 2). However, a similar 5′ clustered organization was not identified in any of the *Dscam* genes from the Mandibulata species of insect, Crustacea or Myriapoda classes, suggesting that it arose after radiation of Mandibulata and *Chelicerata* during the evolution of Arthropoda. Thus, we concluded that the 5′ clustered organization of *sDscam* was *Chelicerata*-specific and conserved throughout *Chelicerata* evolution.

### Origin and lineage-specific expansion of 5**′** clustered *sDscam*

**Figure 2:**
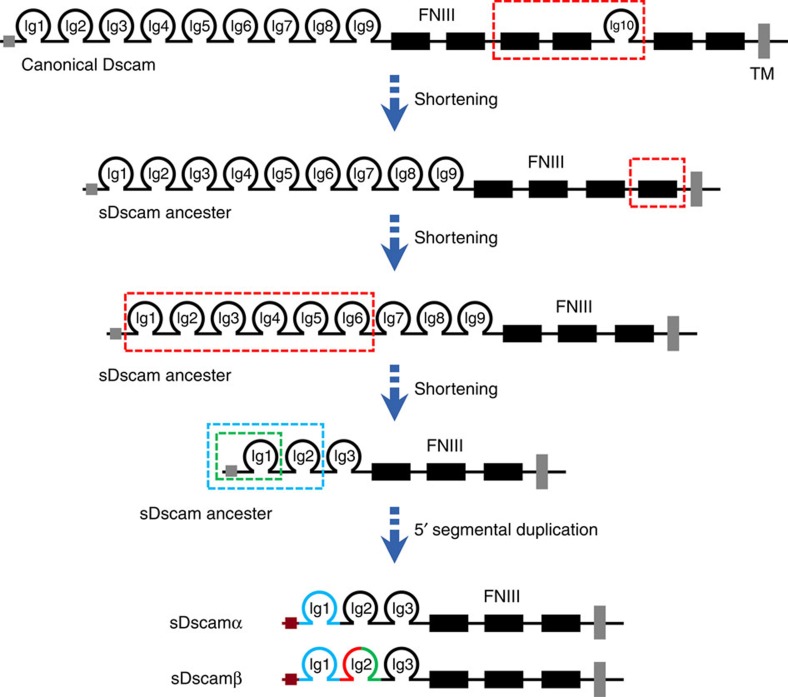
Model of the origins of *sDscamα* and *sDscamβ*. Symbols used are the same as in Fig. 1. The *sDscam* gene may have originated from the sequential shortening and expansion of the Ig and FNIII domains of canonical *Dscam*. First, the ancestral *Dscam* gene underwent sequential shortening of the Ig and FNIII domains of canonical *Dscam* (marked by the red dashed box). Eventually, a shortened *Dscam* evolved in the ancestral gene. This *sDscam* ancestor was followed later by 5′ segmental duplication to create two or more tandemly arrayed cassettes. The duplication unit may have included both exons 1–2 encoding an Ig domain or exons 1–4 encoding 2 Ig domains and their promoters (green or blue dashed boxes). Thus, various isoforms with diverse Ig1 (sDscamα) and Ig1–2 (sDscamβ) were generated by combining alternative promoters with alternative splicing.

How the 5′ clustered organization of the *sDscam* gene arose was investigated next. Following a comprehensive comparative analysis of *Dscam* sequences from arthropod species (Supplementary Fig. 3), it was speculated that the *sDscam* gene might have originated from the sequential shortening and expansion of the Ig and FNIII domains of canonical *Dscam* (Fig. 2, Supplementary Fig. 4a). First, the ancestral *Dscam* gene underwent the loss of FNIII3–4 and Ig10 domains before the divergence of Arachnida and Merostomata. This is supported by the fact that *Dscam* genes lacking the FNIII3–4 and Ig10 domains are present in all *Chelicerata* species investigated (Supplementary Fig. 3). The further loss of the FNIII domain proximal to the transmembrane domain was followed later by the loss of the coding region encoding the N-terminal Ig1–6 domains (Fig. 2; Supplementary Fig. 4a). Eventually, a shortened *Dscam* evolved in the ancestral gene. Second, this shortening was followed later by 5′ segmental duplication to create two or multiple tandemly arrayed cassettes. The duplication unit may include both exons 1–2 encoding an Ig domain or exons 1–4 encoding two Ig domains and their promoters (green or blue dashed box, Fig. 2; Supplementary Fig. 4a). Moreover, phylogenetic analysis indicated that these clustered cassettes were more similar to each other than to the variable cassettes from other species (Supplementary Figs 5 and 6), suggesting that the variable cassettes were expanded in a species-specific manner.

Notably, the genome analysis indicated that most *sDscam* genes tended to be clustered in *Chelicerata* (Supplementary Fig. 4b). For example, three *sDscam* genes clustered in the *T. urticae* genome, of which *sDscamβ2* and *sDscamβ3* were only 4 kb apart and in the same orientation. These findings strongly suggest that *sDscam* gene clusters result from lineage-specific duplications. Together, these results demonstrate that 5′ cassette tandem duplication, combined with gene duplication, jointly shaped the large lineage-specific repertoire of sDscam isoforms in *Chelicerata*.

### Expression patterns of *sDscam* variable cassettes

**Figure 3:**
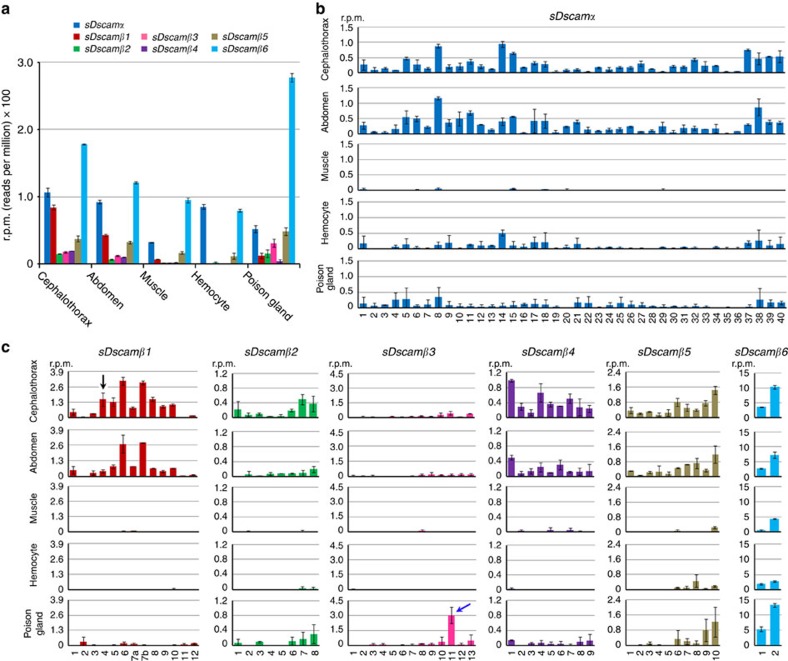
Expression analysis of 5′ variable exons of *M. martensii sDscam*. (**a**) Relative expression levels of *sDscamα* and *sDscamβ1–6* transcripts in different tissues. The expression level for each transcript is shown as reads per million (r.p.m.) of its corresponding constitutive exons. Data are expressed as a percentage of the mean±s.d. from two independent experiments. (**b**) The relative inclusion frequency of the *sDscamα* variable exon in different tissues. Alternative exon 2 was selected to calculate the level of expression. (**c**) The relative frequency of the variable exon clusters of *sDscamβ1–6*. Variable cassette 4 of *sDscamβ1* was abundantly expressed in the cephalothorax (shown as the black arrow), but was barely detectable in other tissues. *sDscamβ3* variable cassette 11 was abundantly expressed in the poison gland (shown as the blue arrow), but was barely detectable in other tissues. The 25-nt fragmented RNA-seq data sets were mapped to calculate the relative expression level. These results based on 25-nucleotide (nt) mapping were consistent with those based on 50-nt mapping, except for some very lowly expressed tissues (Supplementary Fig. 7).

To determine the expression profiles of the variable cassettes in *M. martensii sDscam*s, paired-end sequencing of poly(A)-tailed transcripts was performed on five dissected adult tissue samples, including the cephalothorax, abdomen, muscles, haemocytes and poison glands. RNA-seq reads were mapped to the genome sequence of *sDscam*s as described above. Based on the RNA-seq data of constitutive exons, the *sDscamα* and *sDscamβ1–6* transcripts were differentially expressed (Fig. 3a). The *sDscamα* and *sDscamβ1–6* transcripts were expressed at much higher levels in the cephalothorax than in the abdomen, muscles and haemocytes (Fig. 3a; Supplementary Fig. 7a). This is largely consistent with previous studies in which *Dscams* were highly expressed in neural tissues^13^,^27^. Notably, *sDscamβ3*, *sDscamβ5* and *sDscamβ6* transcripts were expressed at maximum levels in the poison glands. It would be of interest to know whether the *sDscam* isoform diversity contributes to immune protection, as previously reported for *Dscam1* isoforms in insects^27^. Transcriptional signals were detected for almost all of the 5′ variable exons of *sDscamα* and the six *sDscamβ* genes in at least one of the tissues of *M. martensii* (Fig. 3b,c; Supplementary Fig. 7b,c). For each *sDscam* gene, the relative abundance of isoforms differed markedly among the variable exons. For example, the most abundant 10 *sDscamα* isoforms accounted for 54.7% and 52.5% of all reads from the cephalothorax and abdomen, respectively (Fig. 3b,c). Interestingly, the variable cassettes most distal to the constitutive exons tended to occur less frequently in all tissues for all *sDscam*s, except for *sDscamβ4*. In *sDscamβ2–3* and *sDscamβ5–6*, the inclusion frequency of a variable exon largely correlated with its proximity to the first constitutive exon (Supplementary Fig. 8a–d).

Several significant differences existed in the expression profiles of various *sDscam* variable cassettes in different tissues. The 5′ variable exon usage in *sDscamβ1–5* showed moderate to dramatic changes in different tissues, whereas differences in the *sDscamα* cassettes were relatively modest (Fig. 3b,c). Most of the 5′ variable exons of *sDscamα* were expressed in the cephalothorax, abdomen, haemocytes and poison glands. Nonetheless, only a subset was lowly expressed in the muscles (Fig. 3b). Similarly, most of the 5′ variable exons of *sDscamβ1–6* could be detected in the cephalothorax, abdomen and poison glands, while only a subset was expressed in the haemocytes and muscles. Variable cassette 4 of *sDscamβ1* was abundantly expressed in the cephalothorax, but was barely detectable in the abdomen (Fig. 3c; Supplementary Fig. 7c,d). *sDscamβ3* variable cassette 11 was abundantly expressed in the poison gland, but was barely detectable in other tissues (Fig. 3c; Supplementary Fig. 7c,d). These data indicate that the selection of 5′ variable exons of *sDscamα* and *sDscamβ* is differentially regulated in different tissues.

### Variable cassettes are preceded by promoters

**Figure 4:**
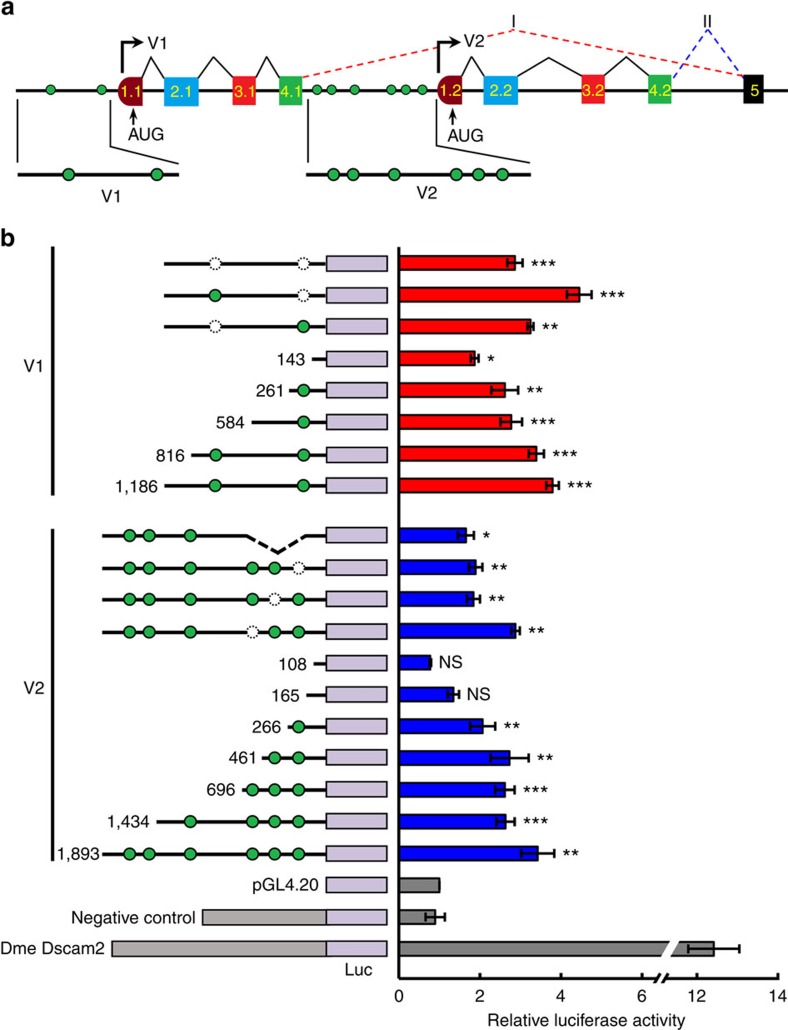
Each variable cassette preceded by a promoter in *sDscam*. (**a**) A schematic diagram of the expression of variable cassettes in *M. martensii sDscamβ6*. Symbols used are the same as in Fig. 1. Potential promoter elements (PPE) are shown as green circles. (**b**) Analysis of *sDscam* variable cassette promoter in the reporter assays. A portion of the sequence immediately preceding a given variable cassette was cloned into a luciferase reporter construct and subsequently transfected into *Drosophila* S2 cells. The luciferase vector containing the *Drosophila Dscam2* promoter or intronic sequence of *sDscamβ6* served as positive and negative controls, respectively. Schematic diagrams of mutants with the indicated sizes are depicted on the left. The deleted PPEs are shown as dashed circles. Data are expressed as a percentage of the mean±s.d. from three independent experiments. *P<0.05, **P<0.01 and ***P<0.001 (Student's *t*-test, two-tailed); NS, not significant.

To clarify the mechanisms by which isoforms were generated and regulated from a single *sDscam* gene locus, it was ascertained whether the *sDscam* genes applied a similar strategy to that in vertebrate *Pcdhs*, with the alternative use of a separate promoter upstream of each first exon of a variable region^19^,^20^. In *Pcdhs*, each first exon is preceded by a promoter and produces a transcript in which the first exon is spliced to common exons. To determine whether each *sDscam* variable cassette has its own promoter, sequences immediately upstream of the transcription start site of each variable region in *sDscamα* and six *sDscamβ* genes were examined. A rich array of potential promoter elements (PPEs) was predicted to be located upstream of the 5′ end of each variable region (Fig. 4a; Supplementary Fig. 9). Therefore our data suggest that each variable cassette is generally preceded by a given promoter.

Next, we firstly validated the promoter activity of *sDscamβ6*, which contains only two tandemly arrayed variable cassettes. To this end, a ∼1.0–2 kb DNA fragment preceding the variable V1 and V2 cassettes was fused to luciferase in an expression vector. As shown in Fig. 4b, both constructs displayed significant promoter activity in transient transfection reporter assays in *Drosophila* S2 cells. This indicates that these predictable promoter sequences are sufficient to direct the reporter expression of heterologous cells. To determine the minimal DNA sequence requirements for promoter activity, a series of deletion constructs was tested. Promoter function was not significantly diminished by truncations to ∼300 bp (Fig. 4b). Moreover, promoter activity was only partially reduced by disruption of a given PPE, suggesting that it resulted from the combinatorial interaction of multiple PPEs, including those beyond the prediction capabilities of the program, which was based on distantly related species. Together, these results indicate that the transcription of individual variable cassettes is under the control of a distinct promoter upstream of each variable exon.

### High splicing complexity across the 5′ variable regions

**Figure 5:**
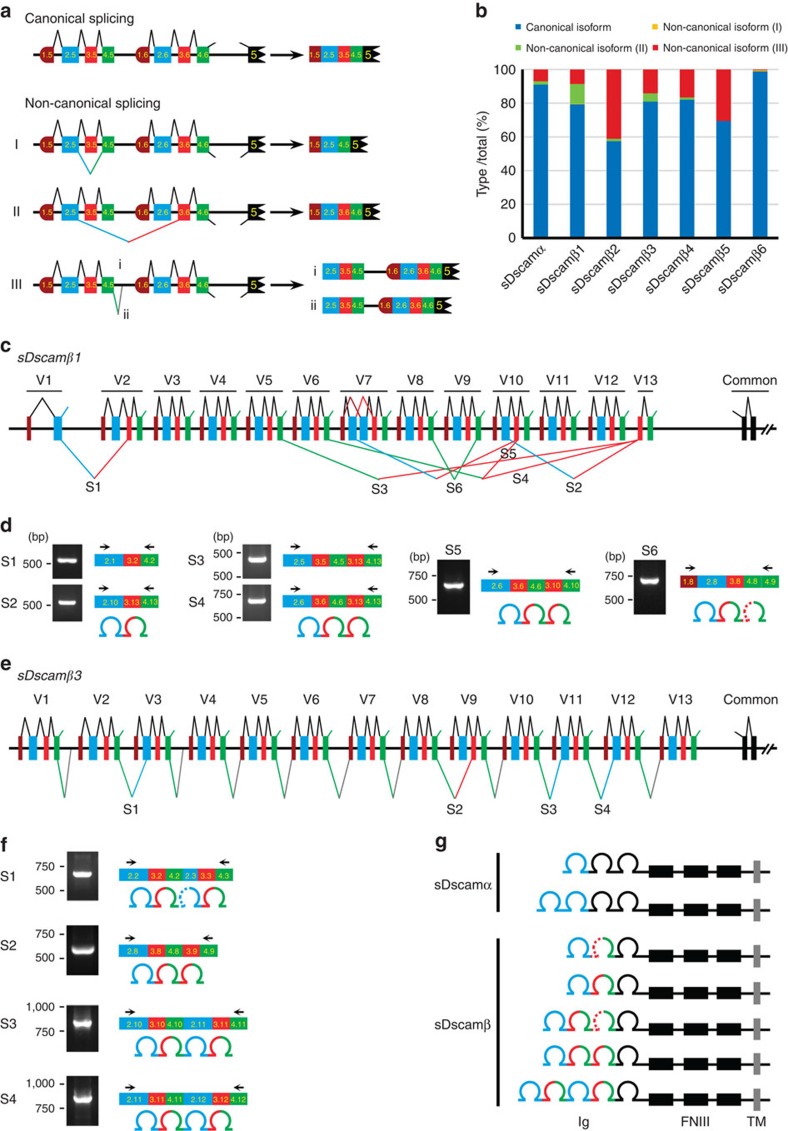
Highly complex combinations of *sDscam* 5′ variable exons. (**a**) Schematic diagram for splicing patterns of the 5′ variable exons. Symbols used are the same as in Fig. 1. Canonical splicing isoforms were joined in neighbouring junctions in variable cassettes, according to the previous ‘cap-proximal splicing' model^19^. Non-canonical splicing isoforms included: (I) splicing isoforms from the same cassette with either exon 2, 3 or 4 skipped; (II) splicing isoforms that contained variable exons from tandem cassettes; as well as (III) the isoforms that contained within-cassette introns. (**b**) Quantification of the canonical and non-canonical splicing isoforms. (**c**) Schematic diagram of the splicing patterns of the 5′ variable exons in *M. martensii sDscamβ1*. Splice isoforms within a single tandem cassette are shown as a black line above the gene structure diagram, while splice isoforms from different tandem cassettes are represented below by coloured lines. (**d**) Alternative splicing junctions from different cassettes were validated using reverse transcription–PCR (RT–PCR). Due to the low expression of *sDscam* variable exons, nested PCR was necessary to amplify the products; only the primers used in the second PCR are depicted and same in panels below. The RT–PCR products were confirmed by cloning and sequencing. These experiments revealed the splicing of multiple cassette variants from different tandem cassettes. (**e**) Splicing patterns of the 5′ variable exons in *sDscamβ3*. (**f**) RT–PCR was used to detect alternative splice isoforms in *sDscamβ3*. (**g**) A summary of several types of isoforms with distinct Ig numbers generated by alternative splicing.

Inconsistent with the presence of a large first exon in the clustered *Pcdh* gene^4^, a cassette repeat composed of two or four exons was identified in the clustered *sDscam* gene. This raised the question of how these variable exons were combined into distinct mRNA isoforms, particularly because the exclusion or multiple inclusions of exons 2, 3 or 4 variants would not result in a frameshift. To explore this, we defined exon junctions based on a total of 0.7 billion RNA-seq reads from different tissues. At least 264 distinct exon junctions were detected, 249 of which were joined neighbouring junctions in single tandem cassettes. This suggests that most isoforms could be made through joining neighbouring junctions in variable cassette regions. Moreover, we detected a small fraction of isoforms from the same cassette with either exon 2, 3 and/or 4 skipped. In these cases, the variable exon skipping resulted in an incomplete Ig domain (that is, the *sDscamβ6* variable exon 3.1) (Fig. 5a). This abnormal splicing is analogous to the skipping of *Dscam* exon 4 variants, which results in a partial Ig2 domain and is likely to be biologically relevant^28^. In addition, we detected other non-canonical splicing isoforms that contained variable exons from different tandem cassettes, as well as the isoforms containing within-cassette introns (Fig. 5a). Based on the exon junctions from the RNA-seq data, we estimated that ∼10–40% of isoforms resulted from non-canonical splicing in most sDscam genes, which showed differential expression in various tissues (Fig. 5a,b; Supplementary Fig. 10a,b). Taken together, these data indicate that *sDscams* have potentially complex splicing patterns at the 5′ variable regions.

Given the low expression of a considerable number of *sDscam* variable exons, we systematically examined the possible exon combinations derived from different tandem cassettes using a nested reverse transcription–PCR (RT–PCR) approach. Several unexpected types of splice isoforms were detected. One type of isoform was produced by combining exons from different tandem cassettes, which encoded 2 Ig domains identical to the canonical isoform from a single cassette. For example, *sDscamβ1* exon 2.1 could be spliced with the downstream variable exon 3.2, while variable exon 3.13 could be spliced with the upstream variable exon 2.10 (Fig. 5c,d). Surprisingly, *sDscamβ1* variable exon 3.13 could be spliced with the upstream variable exons 4.5 and 4.6, and the resulting variable region of the mRNA isoform encoded 3 Ig repeats (Fig. 5c,d). Moreover, *sDscamβ3* variable exon 4.10 could be spliced with the downstream variable exon 2.11, and the resulting variable region of the mRNA isoform encoded 4 Ig repeats. Furthermore, other distinct types of variable 3′ isoforms were detected (Fig. 5e,f). Similar results were obtained for other *sDscamα* and *sDscamβ* genes (Supplementary Fig. 11). Together, these results show that the multi-exon repeat architecture of *sDscams* can increase not only Ig sequence diversity but also Ig number plasticity (Fig. 5g).

### Cap-proximal and downstream exons splice to a constant exon

**Figure 6:**
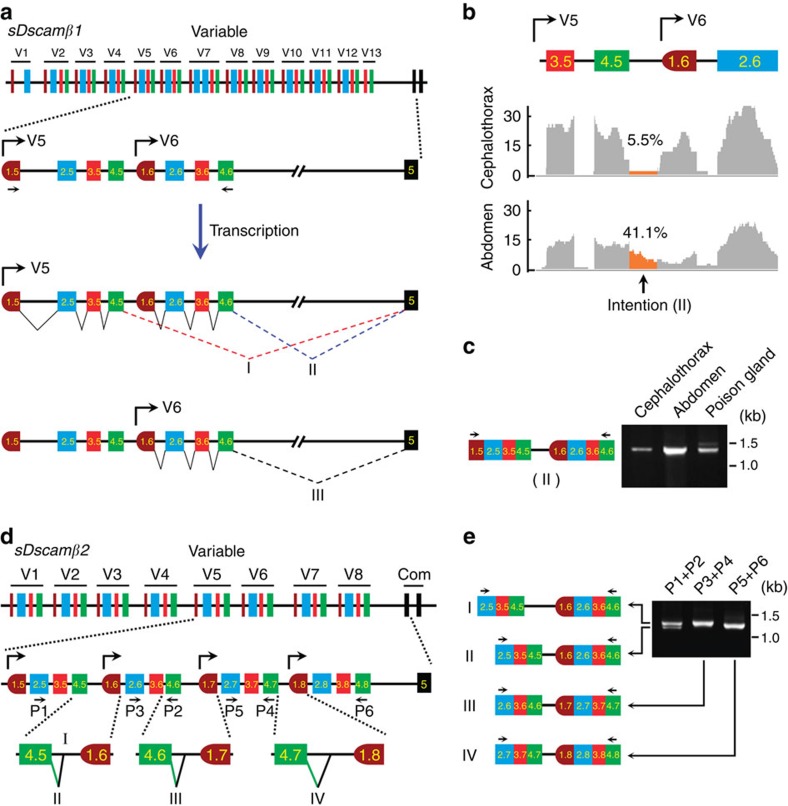
The retention of intron sequences immediately downstream of the last variable exon of each cassette. (**a**) Schematic diagram of *sDscamβ1* isoform expression. Symbols used are the same as in Figs 1 and 4. The expression of the specific combination of *sDscam* isoforms was achieved by alternative promoter activation, followed by alternative splicing. When *sDscamβ1* was transcribed by a V5 promoter, both V5 and the downstream V6 cassette may have been spliced into the constant exon 5. The positions of the PCR primers are indicated. (**b**) Intron retention downstream of the 5′ splice site of the variable cassette (V5) in *sDscamβ1* mRNA reads. Intron retention was much more abundant in the abdomen than in the cephalothorax. The 25-nt fragmented RNA-seq data sets were mapped to calculate the intron retention rate. Because of the low expression of the V5 and V6 isoform in the muscles, haemocytes and poison glands (Fig. 3c), the images of these RNA-seq reads are not shown. (**c**) RT–PCR analysis of V5 and V6 isoform expression. (**d**) Schematic diagrams of expression of *sDscamβ2* isoforms. Different types of splice isoforms are indicated by the symbol "I, II, III, IV". (**e**) RT–PCR was used to detect isoform expression. These experiments revealed the splicing of multiple adjacent cassette variants. Due to the low expression of *sDscam* variable exons, nested PCR was necessary to amplify the products; only the primers used in the second PCR are depicted. The PCR products were confirmed by cloning and sequencing.

Finally, we examined how variable exons were spliced after transcription by alternative promoters. Although previous studies suggested that only the cap-proximal variable exon was joined to the first constant exon in vertebrate *Pcdh*s^4^,^19^,^20^, this hypothesis had not been validated experimentally due to the large size (∼200 kb in the variable regions) and complexity of the clustered *Pcdh*s. Surprisingly, we found that abundant intron sequences immediately downstream of the last variable exon of each cassette were frequently retained in the RNA-seq data, while cassettes within introns were exclusively spliced out (that is, *sDscamβ1* V5, Fig. 6a,b). Interestingly, the extent of this retention differed in different tissues (Fig. 6b; Supplementary Fig. 10b). The frequent occurrence of this unusual intronic retention might be a result of the splicing of the variable exons immediately downstream of the cap-proximal cassette to the constant exon (type II; Fig. 6a). Taken together, we propose that not only the cap-proximal, but also the downstream variable exons spliced to the constant exon.

Next, a more sensitive assay was designed that used primers in exons 1.5 and 4.6 to validate the findings above (Fig. 6a). It was hypothesized that if the downstream variable cassette 6 (V6) could be spliced into the constant when *sDscamβ1* was transcribed under the control of the V5 promoter, then one mRNA isoform should be produced containing the two neighbouring variable cassettes (V5 and V6) without a within-cassette intron, but with the between-cassette sequence (type II, Fig. 6a). The presence of this mRNA isoform was confirmed by RT–PCR and sequencing (Fig. 6c). A similar mRNA isoform was detected in *sDscamβ2*, although the partial interval sequences between the two neighbouring variable cassettes had been spliced out (Fig. 6d,e). Similar mRNA isoforms were observed in other *sDscamβ* genes (Fig. 5e; Supplementary Fig. 11c–h). Taken together, these observations strongly support our hypothesis that not only the cap-proximal, but also the downstream variable cassettes could splice to the constant exon. This also suggests that the expression of 5′ variable cassettes is not only associated with specific promoter activity, but also with post-transcriptional alternative splicing.

## Discussion

This study identified a novel shortened *Dscam* gene family with tandemly arrayed 5′ cassettes in *Chelicerata*. These *sDscams* had a high sequence similarity to the 3′ region of *Drosophila Dscam1*, but shared striking organizational resemblance to the 5′ variable region of vertebrate clustered *Pcdh*s. Moreover, *sDscam* gene family members tended to be arranged in tandem clusters, much like the vertebrate clustered *Pcdh* genes^4^. Finally, *sDscam*s generally contained separate promoters upstream of each first exon of the variable cassette, as occurs in vertebrate *Pcdh*s^19^,^20^. Hence, our findings have important implications for understanding the functional similarities between *Drosophila Dscam1* and vertebrate *Pcdh*s.

Compared with the large exons in clustered *Pcdh* genes, *Chelicerata sDscam* genes were composed of two to four exons. This tandem multi-exon organization not only expanded the diversity of amino acid sequences, but also enabled Ig structural plasticity. In *Chelicerata sDscams*, additional alternative splicing methods might be employed to expand isoform diversity (Fig. 5). For example, additional isoform diversity could be generated through mutually exclusive splicing of within-cassette duplicated exons (that is, *sDscamβ1* V7; Fig. 5c). Notably, additional sequence and structural diversity could potentially be generated through combining exons from different tandem cassettes. Thus, clustered *sDscams* could potentially achieve much more isoform diversity than the clustered *Pcdh* gene. It is very likely that this more complex organization provides a genetic mechanism for generating higher numbers and additional types of isoforms required for the diverse functions and adaptations in *Chelicerata*.

**Figure 7:**
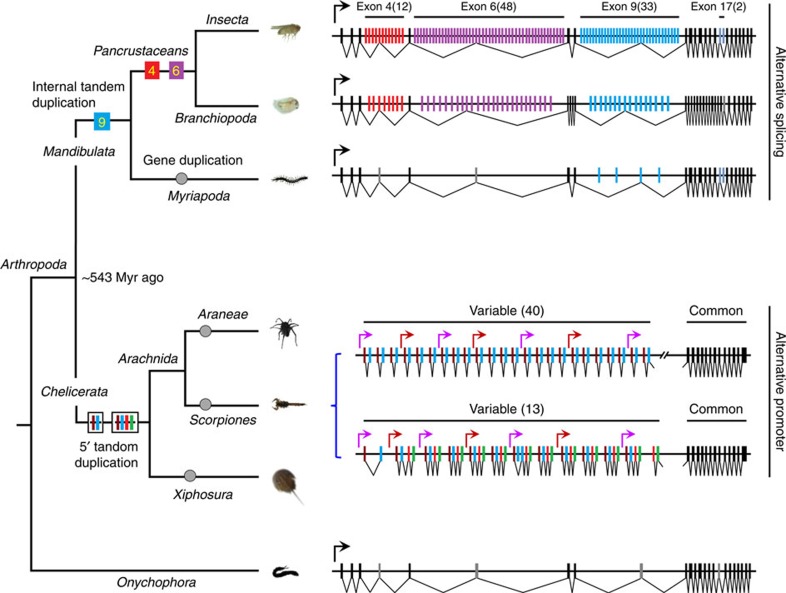
Arthropoda diversify two systems to generate Dscam isoforms. Exons that are arranged in a tandem array or their orthologues are shown in coloured boxes, while the constitutive exons (CE) flanking the duplicated exons or their orthologues are shown in the black box. The introns are represented by the lines and are not drawn to scale. The emergence of internal tandem exon duplication is indicated by the filled squares. The emergence of 5′ cassette duplication is indicated by black line squares. The filled circle represents gene duplication. Extant organization of *Dscam* pre-mRNA and proposed ancestor molecules shown are associated with a cladogram of the phylogenetic relationships in this study^26^. *Dscam1* in *D. melanogaster*, *D. pulex* and *S. maritima* are shown according to previous studies^3^,^25^,^36^. The analysed species and detailed *sDscam* data are shown in Fig. 1 and Supplementary Fig. 2. The number of copies is shown in parentheses.

Phylogenetic analysis of Arthropoda *Dscam* genes revealed that *Chelicerata sDscam* and *Drosophila Dscam1* were classified into different clades (Supplementary Fig. 3), suggesting that they may have converged on the common protein domain diversity from independent origins. Notably, duplication of the Ig7-encoding exon 9 or its orthologues occurred internally or 5′ terminally in all Arthropoda species investigated. This suggests that the diversity of *Dscam1* Ig7 or its orthologues conferred intrinsic structural and regulatory benefits during Arthropoda evolution. Recent studies indicated that Ig7 domain diversity was crucial for the proper function of Dscam1 (refs 6, 8, 10, 12, 13, 14). *Dscam1* generates functionally distinct isoforms through mutually exclusive splicing of internal exons in *Drosophila* (Fig. 7). However, no *Chelicerata Dscam* genes appeared to have a similar arrangement, although a random array of only two alternatives for the *Dscam1* exon 9 orthologue are often observed in *Chelicerata* (that is, *sDscamβ1* V7). In contrast, *sDscam* genes have evolved other mechanisms that serve this function in *Chelicerata*, through a combination of alternative promoter use and alternative splicing (Fig. 7). In this scenario, *Drosophila Dscam1* and *Chelicerata sDscam* represent examples of convergent evolution for isoform diversity.

It is noteworthy that, compared with *Drosophila* Dscam1 and other Dscam proteins from metazoans containing 10 Ig and 6 FNIII extracellular repeats, a single transmembrane segment and a cytoplasmic tail^15^, the *Chelicerata* sDscams reported in this study lacked the N-terminal Ig1–6,10 domains and FNIII3–4, 6 domains present in classical DSCAM. In fact, the Ig domains differed markedly across the immunoglobulin superfamily (IgSF) proteins, ranging from 2 to 10, but with mostly 4 to 5 repeats^29^. Hence, we speculate that such shortened isoforms have important functions. Because *Chelicerata sDscams* share a striking similarity with *Drosophila Dscam1*, and there was a remarkable organizational resemblance to the vertebrate clustered *Pcdh*s, with the latter two proteins both able to mediate self-recognition and self-avoidance, it is reasonable to speculate that *Chelicerata* sDscams have analogous roles in the nervous system.

**Figure 8:**
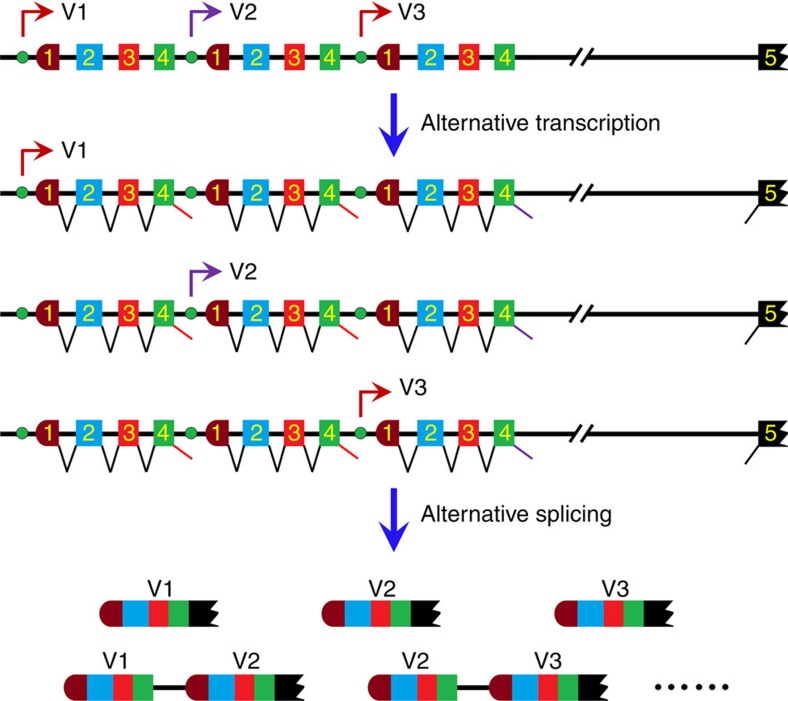
Model of *sDscamβ* isoform expression. Symbols used are the same as in Fig. 1. Each variable cassette was preceded by a promoter. The expression of the specific combination of *sDscam* isoforms was achieved by alternative promoter activation, followed by alternative splicing. When *sDscamβ* was transcribed by a given promoter preceding a variable cassette (V1), both V1 and the downstream variable cassettes (V2, V3) could be spliced into the constant exon 5.

Our results indicated that not only the cap-proximal but also the downstream variable cassettes spliced to the constant exon. Based on this evidence, we propose a mechanistic framework for the selection of tandemly arrayed 5′ variable exons (Fig. 8). This extends and revises a previously proposed model for the mechanism governing the selection of tandemly arrayed 5′ variable regions^4^,^19^,^20^. Interestingly, intron sequences downstream of the variable region exons of *Pcdh*s were frequently contained in complementary DNA (cDNA) in independently derived cDNA libraries, which were previously assumed to be truncated mRNA isoforms or correspond to *trans*-splicing precursors^4^. Considering the similarity of the 5′ gene structure of *Chelicerata sDscams* and vertebrate *Pcdh*s, we speculate that these unusual intron-containing cDNAs might be a consequence of the variable exons downstream of the cap-proximal exons spliced to the constant exon in vertebrate *Pcdh* genes. Therefore, our mechanistic framework might be broadly applicable to tandemly arrayed 5′ variable exons in invertebrates and vertebrates.

The selection of tandemly arrayed 5′ cassettes was highly regulated by a variety of mechanisms at both the transcriptional and post-transcriptional levels. Previous studies indicated that expression of the corresponding *Pcdh* mRNA might correlate with specific promoter activity^19^,^20^. Because *sDscam* was under the control of a distinct promoter upstream of each variable cassette, *Chelicerata sDscams* should be regulated by a similar mechanism. Second, the 5′ splice site strength might have an effect on the selection of the variable exon. In general, the variant inclusion largely correlated with the strength of the 5′ splice site, but decreased with distance from the 3′ splice site of the first constitutive exon^30^. Based on the correlation of the inclusion frequency of a variable exon with its proximity to the first constitutive exon in *sDscamβ2*–*3* and *sDscamβ5*–*6*, it seems that distance had some effect on the inclusion, at least for some genes. This was possibly due to higher levels of pre-mRNA for the proximal exons of the first constitutive exon present after transcription under multiple promoters. Finally, the selection of variable cassettes could easily be overridden in a developmental- or tissue-specific manner by the expression of specific activator- and repressor-binding proteins. Thus, the outcome of the variable exon results from multiple mechanisms acting in an overlapping manner.

## Methods

### Annotation and identification of *Dscam*s

The sequences of the *Dscam* genes from the Scorpione *M. martensii*, the Araneae *S. mimosarum*, the Ixodoidean *I. scapularis* and *T. urticae*, and the Merostomatan *L. polyphemus* have been annotated through BLAST searches, using the annotated *Dscam* sequence of the most closely related organism and confirmed by available genome annotation and phylogenetic analysis (http://blast.ncbi.nlm.nih.gov/Blast.cgi; http://flybase.org/blast/, Supplementary Table 1). Gaps in the *Dscam* sequences for *M. martensii* were closed by PCR and sequencing. Genomic DNA was isolated from *M. martensii* (a gift from Zhijian Cao) using a QIAamp DNA Kit (Qiagen, Hilden, Germany). PCR was performed using primers designed against genomic sequences. Amplification products were cloned into the pGEM-T Easy Vector (Promega, Madison, WI, USA) for sequencing. Primer sequences are available on request. All *Dscam* homologues were analysed by classifying into families and predicting domains with InterPro^31^ (http://www.ebi.ac.uk/interpro/).

### RNA-seq

Five tissues (cephalothorax, abdomen, poison gland, haemocyte and muscle) from an *M. martensii* adult and the whole body of a *L. polyphemus* adult were collected for RNA preparation. RNA library construction and paired-end RNA-seq were performed by LC Sciences (Houston, TX, USA). Briefly, total RNA was extracted using TRIzol reagent (Invitrogen, Carlsbad, CA, USA) according to the manufacturer's instructions. The total RNA quantity and purity were analysed using a Bioanalyser 2100 and RNA 6000 Nano LabChip Kit (Agilent, Santa Clara, CA, USA) with RNA integrity number >7.0. For the RNA-seq experiment, ∼10 μg of total RNA was subjected to enrichment of the poly(A)-tailed mRNAs with poly(T) oligo-attached magnetic beads (Thermo Fisher Scientific, Waltham, MA, USA). After purification, the mRNA was fragmented into small pieces using divalent cations under elevated temperature. Then the cleaved RNA fragments were reverse transcribed to produce the final cDNA library according to the instructions in the mRNA-seq sample preparation kit (Illumina, San Diego, CA, USA). The paired-end RNA-seq was performed on the Illumina Hiseq 2500 platform (Illumina) following the vendor's recommended protocols.

### Analysis of RNA-seq data

The RNA-seq reads were *de novo* assembled to obtain transcripts of *M. martensii* and *L. polyphemus* using Trinity^32^ (https://github.com/trinityrnaseq/trinityrnaseq/wiki) with the default parameters. Transcripts sharing high sequence similarity were assigned to a cluster based on the default parameter settings of Trinity. For a cluster, the longest transcript was designated as the unigene of the cluster. The unigenes were functionally annotated based on sequence similarity at the protein level. Specifically, by using BLASTX (*E*-value<0.00001), the protein sequences translated from the unigenes were searched against the protein databases, including the NCBI non-redundant protein database, SwissProt, Kyoto Encyclopaedia of Genes and Genomes (KEGG) and Clusters of Orthologous Groups (COG) of proteins. The ends most 5′ of the *sDscam* unigenes were analysed for their potential transcription start sites, some of which were further verified by 5′ RACE.

Tophat^33^ (http://ccb.jhu.edu/software/tophat/index.shtml) was used for RNA-seq mapping, the results of which were visualized using integrative genomics viewer (IGV)^34^ (http://www.broadinstitute.org/igv/). Considering the similarity among exon duplicates, the RNA-seq reads were split into 25- and 50-nucleotide (nt) fragments, which were mapped to calculate the expression levels of variable exons. Furthermore, to eliminate influences on calculations of the expression levels from identical sequence regions among exon duplicates, the 25- and 50-nt fragments with multiple loci were correctly allocated by referring to the mapping results of the full-length RNA-seq data sets. The correlation coefficient was calculated between the 25- and 50-nt mapping results. Similarly, to analyse the intron retention rate, both the 25- and 50-nt fragmented RNA-seq data sets were utilized to calculate the expression levels of the exon and neighbouring intron.

An in-house computational program was developed to search for sequencing evidence supporting the exon–exon junctions. First, exonic sequences covering all of the possible junctions between the variable exons were created. We used 10 positions from each exon in a pair to assign a given read to an exon–exon junction. For example, the 230-nt exonic sequences included 115-nt upstream and 115-nt downstream of the junction for 125-nt RNA-seq reads. Second, all of the RNA-seq reads were mapped onto the exonic sequences created above, and the perfectly mapped RNA-seq reads covering exon–exon junctions were retained. A similar analysis was performed for 25 positions from each exon in a pair to determine the correlation with the results based on 10 positions. In addition, a similar method was used to analyse the exon-intron junction of the isoforms containing within-cassette introns.

### RT–PCR

Total RNA was isolated using an RNeasy Mini Kit (Qiagen). Total RNA was reverse transcribed using SuperScript III RT (Invitrogen) with oligo(dT)15 primer, and the resulting single-stranded cDNA product was treated with DNase I at 37 °C for 30 min. The PCR was implemented with an initial denaturing at 95 °C for 3 min, followed by 35 cycles of denaturing at 95 °C for 45 s, annealing at 55 °C for 50 s, and extension at 72 °C for 2 min and 10 s, followed by a final extension at 72 °C for 10 min. The products of the PCR or the RT–PCR were purified and cloned into the pGEM-T Easy Vector and transformed into JM109 competent cells. Sequencing of individual clones was carried out using an automatic DNA sequencer. In some cases, nested PCR was necessary to amplify the products. Primer sequences are listed in Supplementary Table 2.

### Phylogenetic analysis

The alignment of specific regions between species was performed using the ClustalW2 program (http://www.ebi.ac.uk/clustalw/index.html). Full-length variable region coding sequences were translated, and the resulting polypeptides were aligned. The genetic distances for each gene were estimated with MEGA 6.0 (ref. 35).

### 5′ RACE analysis

The 5′ RACE analysis was performed according to the 5′ RACE Kit (Invitrogen) protocol and using the reagents from the kit. Total RNA was extracted from adult *M. martensii* cephalothoraxes using TRIzol Reagent (Life Technologies, Carlsbad, CA, USA). The RNA was subjected to reverse transcription using SuperScript II at 42 °C for 50 min, and incubated at 70 °C for 15 min to terminate the reaction. RT–PCR was carried out under the following cycling conditions: an initial denaturation of 2 min at 94 °C followed by 30–35 cycles of denaturation at 94 °C for 30 s, annealing at 55–60 °C for 30 s and extension at 72 °C for 30 s, with a final extension at 72 °C for 10 min.

### Promoter activity analysis

The promoter distribution was predicted using the Berkley Drosophila Neural Network Promoter program (http://www.fruitfly.org/seq_tools/promoter.html). To assay the promoter activity for *M. martensii*, the corresponding DNA sequences immediately preceding the translational start site of *sDscamα* and *sDscamβ* were cloned into a pGL4.20-Fluc reporter vector (Promega). For *sDscamβ6* V1 and V2, site mutagenesis was performed to disrupt the predicted core promoter elements based on the schematic diagrams of minigene constructs (Fig. 4). The intron sequence in the common region of *sDscamβ6* was cloned as a negative control. The pGL4.20 vector was used as a blank control. The promoter DNA sequence immediately preceding the translational start site of *D. melanogaster Dscam*2 was cloned as a positive control. All constructs were confirmed by sequencing. *Drosophila* S2 cells were co-transfected with the pGL4.20-Fluc reporter plasmid and the *tubulin* promoter-Rluc reporter plasmid (a gift from Wanzhong Ge) with Lipofectin (Invitrogen) according to the manufacturer's instructions. Cells were lysed 48 h post transfection to measure the activity of firefly and Renilla luciferase according to the Dual-Luciferase Reporter Assay System (Promega). The mean and s.d. values were determined for each construct based on three independent transfections. The error bars were calculated from the average of three independent experiments in this study. The significance of differences was determined by a two-tailed Student's *t*-test and *P<0.05, **P<0.01 and ***P<0.001 were taken to indicate statistical significance.

## Additional information

**Accession codes:** The RNA-seq data were deposited into NCBI SRA (Sequence Read Archive; http://www.ncbi.nlm.nih.gov/sra/) (accession numbers: SRX1319503, SRX1319674, SRX1319813, SRX1319876 SRX1319877, and SRX1323743). The *Dscam* gene sequences were deposited into GenBank with accession numbers KT932388-KT932417; KU378204-KU378205.

**How to cite this article:** Yue, Y. *et al*. A large family of *Dscam* genes with tandemly arrayed 5′ cassettes in *Chelicerata*. *Nat. Commun.* 7:11252 doi: 10.1038/ncomms11252 (2016).

## Supplementary Material

Supplementary Information Supplementary Figures 1-11, Supplementary Tables 1-2 and Supplementary References
