# Supplementary material for: A large family of Dscam genes with tandemly arrayed 5′ cassettes in Chelicerata
Source: Nat Commun. 2016 Apr 15;7:11252. doi: 10.1038/ncomms11252 (PMC4835542; doi:10.1038/ncomms11252)
Supplement: Supplementary Information — Supplementary Figures 1-11, Supplementary Tables 1-2 and Supplementary References [file ncomms11252-s1.pdf]

Supplemental information

sDscama-V1
gcaacgtaatatttcggaaaattctcgctagaggtcctcatcggaatgtttctttgctttatcacgtgtatcttaaatgtatgtttttggaaagttctcattgctaatgctgcggtaccaccagaaattaagccattttatt…
A T - Y F G K F S L E V L I G M F L C F I T C I L N V C F W K V L I A N A A V P P E I K P F Y …

sDscama-V2
atgatgtaattatttttgaatggtctacaagaggtcgccactaaaatgtgtttttggatggtaaagaaattaatccttattcttcatttatggagttcgagtaacgcaagtggtagttctcctccaaaaataatgccttttc…
M M - L F L N G L Q E V A T K M C F W M V K K L I L I L H L W S S S N A S G S S P P K I M P F …

sDscama-V3
ggtatctaaaaatttctttcgtttacaatggataatagaaagagatttaaaggagagtctgcaagcattgctatgaaaatgtctcaaattctgaccaatttttatttgtttacgtcgcatggctatgtaaaactgcaatcg…
G I - K F L S F T M D N R K R F K G E S A S I A M K M S Q I L T N F L F V Y V A W L C K T A I …

sDscama-V4
aagctgtgacttgcacagacaatgatattatatctgtttatatttttaacgtttcctctagtgaagccatttcactacctcaaattaagccatttatttttcaacgaccattcaagaagggcaaatggaacaaataacat…
K L - L A Q T M I L Y L F I F L T F S S S E A I S L P Q I K P F Y F S T T I Q E G Q M E Q I T …

sDscama-V5
caagagtagacgttagaggtcagcactaagatgtccatcacttggaaaattatttatttaacgcatatttcatggatttcgggaatttttactagtactattcctcctcaaattaaaccgttttacttctcaaaaaacgtag…
Q E - T L E V S T K M S I T W K I I Y L T H I S W I S G I F T S T I P P Q I K P F Y F S K N V …

sDscama-V6
ttcaggtgagatttcgatatatatttcatttcagtgacttaaatagtttcggatagttaaaattatatttcagataatatatttattcttgcagctcaattttataaacatctcatacagataattgtgatgacatatt…
F R - D F D I Y F H F S D L N S F R I V K I I F Q I I Y F I L A A Q F Y K H L I Q I I V M T Y …

sDscama-V7
tattgataaatttccgatatcatctatcgtttgagatattttatggtacgaatattctcctttgttcaaatacagtagtctactttattgaatattcgccagaggtcgacagcatgatgacgattattagaaaactgtata…
Y - - I F R Y H L S F E I F Y G T N I L L C S N T V V Y F I E Y S P E V D S M M T I I R K L Y …

sDscama-V8
ctttattaatgtggaaagctctgtcgatacaatagtacaaccgtctctttcaaaatggccattctactactatttctctttggaatggcatgtatcacaaatggcataattattccgaaaattaaaccattttattttcat…
L Y - C G K L C R Y N S T T V S F K M A I L L L F L F G M A C I T N G I I I P K I K P F Y F S …

sDscama-V9
atatgatgaactattccgtatacatatctcaatgaagtcgaaagctgtagatataagatttattataaattgctttcgatttaagaattattcaagtaataagtggctttatggagttagttttcctttgtatcgtggttg…
I - - T I P Y T Y L N E V E S C R Y K I Y Y K L L S I L R I I Q V I S G F M E L V F L C I V V …

sDscama-V10
gattttaagtaataatgctagttataatcttttgtgtatcgtgtttaggctgctttaacacgctaattgctgccagtatgcctaaaataaaaccatttcatttcactaaagatattcatataggccagcgagaacaagtaa…
D F - V I M L V I I F C V S C L G C F N T L I A A S M P K I K P F H F T K D I H I G Q R E Q V …

sDscama-V11
gcatattaatctatttgaatttttacagattactacataaaacaatgatgattaatagacataaaagtttagtcattatttattatacgatatatttttcaatgtatatggaaacaccattccgaaaattaaaccatttc…
A Y - S I L N F Y R L L H K T M M I N R H K S L V I I Y Y T I Y F F N V Y G N T I P K I K P F …

sDscama-V12
ttcgtttgaaaatttcaaaatcaaatacagtgaaaaattgtatcaagatagcaatggaaagttttataaagatcaagatgaaaacagttattctgttccttactatatctcattgttttagtaatgttgttcctcctaaaa…
F V - K F S K S N T V K N C I K I A M E S F I K I K M K T V I L F L T I S H C F S N V V P P K …

sDscama-V13
gagtattaagcaatgggagttatttgcacagaaatttaataactgtttgctacttaatatttctctgctgtgtttatggaaacactataccaaaaattaaaccgttccatttcactcaaaatcttcgtgaaggtctgaaac…
E Y - A M G V I L H R N L I T V C Y L I F L C C V Y G N T I P K I K P F H F T Q N L R E G L K …

sDscama-V14
taaatgtaacaaaatgcattaattcagtatttaaataattgcattcgtgtggcaatgttaagttttataaagatcgagatgaaaacattgcttctgttgtttgctatatcctattgttcttgccacacgtctccacctaaaa…
- M - Q N A L I Q Y L N N C I R V A M L S F I K I E M K T L L L L F A I S Y C S C H T S P P K …

sDscama-V15
aattattaaacaatgaaagtttcttggtatagaagttcaataataatttcctgtataatttcattgtgttgcacttatggaaagtctataccgaaaataaaaccattccatttcactcaaaatcttcgtgaaggtcttaaag…
N Y - T M K V S W Y R S S I I I S C I I S L C C T Y G K S I P K I K P F H F T Q N L R E G L K …

sDscama-V16
aatttgtgaatatgtttcaacactaatatgaaaatgcaaacattcgcaaaatacgagtggaagataattatattattccatactgtattcctttgtgtttgtaatatatctccacctaaaataaagccatttcatttcactc…
N L - I C F N T N M K M Q T F A K Y E W K I I I L F H T V F L C V C N I S P P K I K P F H F T …

sDscama-V17
gagttatagtttctcacatttatttggttacttgagttaattttgctaaaacatttatgtattcaaaagtattttaagatgttaatgattgatttcacgaggatcgagatgaaaatattcttcttgctcttgtctgcttcac…
E L - F L T F I W L L E L I L L K H L C I Q K Y F K M L M I D F T R I E M K I F F L L L S A S …

sDscama-V18
aagttttaagctatttgcaaaaaaagtttgattatgatattgtatgatttaatatttgttgttttacttctctttgtggattcctctatttcgattaatccaccaaaaataaaaccatttcaatttactgaaaatgtgaaac…
K F - A I C K K S L I M I L Y D L I F V V L L L F V D S S I S I N P P K I K P F Q F T E N V K …

sDscama-V19
tgttaataactaaatatggagaaacagtattatgtctttcttaatgttttgcttcttcctatagtttttggtttaacgtttccgaaaatcaaacctttccatttactaacgatgttagagaaggtcagagacaacaagtaa…
C - - L N M E K Q Y Y V F L N V L L L P I V F G L T F P K I K P F H F T N D V R E G Q R Q Q V …

sDscama-V20
ccagaatgaaaggcctacataaaaatatctactatatgtagaaatatcatgatgaaagaaatgttactacggtgtttgctaatatttatcgtgtttgcttattcagatggaggaaaatttccaccaaaaatcaaaccatttc…
P E - K A Y I K I S T I C R N I M M K E M L L R C L L I F I V F A Y S D G G K F P P K I K P F …

sDscama-V21
gtttttaatatataatctatagagaactcaaatacgaaattagaatggattattacattgatctgatagcattgatgttggttttcggtttgtccagagccatgattccaccaaaagtcaggccttttcactttaccacag…
V F - Y I I Y R E L K Y E I R M D Y Y I D L I A L M L V F G L S R A M I P P K V R P F H F T T …

sDscama-V23
tttttttaagaaacgtgtaaactatactatgttttaaatagtttattaaggtttaaaatggaagatttcatcaaggtcgaaatactttgttgtcgcttattgtatcacattgtttttgcaatgtttctccacctataatca…
F F - E T C K L Y Y V L N S L L R F K M E D F I K V E I L L L S L I V S H C F C N V S P P I I …

sDscama-V24
ttcagttgatggactcaaatacgaaataaagcaatggattatcatattgatctcgtaacattgcttttgactttcagttggtgtagagccgtgattccaccgaaagtcaaacctttcactttgccacagacataaggattg…
F S - W T Q I R N K A M D Y H I D L V T L L L T F S W C R A V I P P K V K P F H F A T D I R I …

sDscama-V25
tttatataatttaagtggagtttcttaagaaacgtgtatgcgatactatgtttcaaaaagtttaagttgcaaatggcagatttcatcaagatcgagatactttgctgttgcttattgtatcacattgtttttgcagtattt…
F I - F K W S F L R N V Y A I L C F K K F K L Q M A D F I K I E I L L L L L I V S H C F C S I …

sDscama-V26
attcgataaagaactgaaatgcgaattaaggcaatggattataatattgatctggtaatatttctcttggctttcagttggtgtacagccgtgattccaccgaaagtcaagccttttcactttgccacagatataagaattg…
I R - R T E M R I K A M D Y N I D L V I F L L A F S W C T A V I P P K V K P F H F A T D I R I …

sDscama-V27
aacatatgatacttttttcaaaaagtttaaattgcaaatggcagctttcatcaagatcgagatactttgttgttgcttattgtatcacattgtttttgcagtgtttctccacctataatcaaaccatttcatttctcaaaag…
N I - Y F F K K F K L Q M A A F I K I E I L L L L L I V S H C F C S V S P P I I K P F H F S K …

sDscama-V28
tgttaatagctaaacatggaaataatatacggcaatgtctttctcagtttttactttttcctctaactctaggtataacgcttccaaaaatcaaacctttccatttactaacgatgttagagaagggcagagacagcagt…
C - - L N M E I I Y G N V F L S F L L F P L T L G I T L P K I K P F H F T N D V R E G Q R Q Q …

sDscama-V29
agattatagaacaattttcatttcgacttcgttatcttgcgattaatcatgaacacattttgagaagttatctaacctttctgcttttgtctgttcatcaagttgtttatgctcttttgcccccgaagattcgtccttttc…
R L - N N F H F D F V I L R L I M N T F L R S Y L T F L L L S V H Q V V Y A L L P P K I R P F …

sDscama-V30
tgaatataaaaatatgatataactgaaatgaatgaaatgctgctattgtatttatttatgtttgttataacttattcagatgcaggaaaatttctcctaaaattaaaccatttcactttacaaaagatgtacaaagtggag…
- I - K Y D I T E M N E M L L L Y L F M F V I T Y S D A G K F S P K I K P F H F T K D V Q S G …

sDscama-V31
caatgataaactcaaatacgaaacaaaacaatggattgttatattgatctgatagcattactattgactctcaattttttgcgaagctgtgactccaccgaaagtcaagccttttcattttaccacagacataagaatgggtc…
Q - - T Q I R N K T M D C Y I D L I A L L L T L N F C E A V T P P K V K P F H F T T D I R M G …

sDscama-V32
tgtatatgatatgttttaaatagtttactaaacgaacaaatggaaaatttcatcaagatcgaaatactttttattgttgcttattgtatcacattgtttttgcagtgtttctccacctgtaatcaaacagtttcatttctcga…
C I - Y V L N S L L N E Q M E N F I K I E I L L L L L I V S H C F C S V S P P V I K Q F H F S …

sDscama-V33
tgctaataactaaacatggaaatacatggcgatatctttcttggtattctggttcttcctctaactctcggtataacgcttccgaaaatcaaacctttccatttactaacgatattagagaaggacagagacagcagttaa…
C - - L N M E I H G D I F L G I L V L P L T L G I T L P K I K P F H F T N D I R E G Q R Q Q L …

sDscama-V34
agattataaaacatttttcatttcgacttcatcatcttacggttaatcatgaacacattttttgagaaattgttatctaacttgtctactttttgtctgttgttcactttgttcatgctcttttgcctccgaagattcgtcctt…
R L - N I F H F D F I I L R L I M N T F L R N C Y L T C L L L S V V H F V H A L L P P K I R P …

sDscama-V35
cgaaattaaacgatggatgattatatttatctgatgatatttctgatggctttcagtttatgtagaggtataacatttccgaaaatcaaacctttccattttactaatgatgttagagaaggtcagagacaacaagtaactt…
R N - T M D D Y I Y L M I F L M A F S L C R G I T F P K I K P F H F T N D V R E G Q R Q Q V T …

sDscama-V36
ccagaatgaaaggcctacataaaaatacttactatgtgtagaaacatcatgataaaagaaatactgttaagatgtttgctaatatttattgtgtttgcttattcagatggaggaaaatttcctccaaaaatcaaaccatttc…
P E - K A Y I K I L T M C R N I M I K E I L L R C L L I F I V F A Y S D G G K F P P K I K P F …

sDscama-V37
gtttttaaagtataatctgtagagaactcaaatacgaaattaggatgaattattacattgatctgatagcattgctgttgactttcggtttgtccaaagctgtgattccaccgaaagtcaagcattttcattttgccacag…
V F - S I I C R E L K Y E I R M N Y Y I D L I A L L L T F G L S K A V I P P K V K H F H F A T …

sDscama-V38
atacagtgaatttacttatcatgtatttatcgctggatatcaaaatactaatcatatcttcaataactatatttcatgttttgggcaacatttcgccaaaaattaagccattttatttctcaaaagacgtacaagaaggag…
I Q - I L L I M Y L S L D I K I L I I S S I T I F H V L G N I S P K I K P F Y F S K D V Q E G …

sDscama-V39
ttataataattttttaatgatgaatggttattacctaattatcttttggggtttgcacatatctgttgcaataaatataccaagaatcaaaccttttcactttactaaagacatcaaagaaggtgagagagaacagattattt…
L - - F L M M N G Y Y L I I F W G L H I S V A I N I P R I K P F H F T K D I K E G E R E Q I I …

sDscama-V40
gtgctgtagttaaaattgatcatgctgatgattcacgtagtacactttaccaaatatggtttaaatccgcctaaaataaaacaatttcattttctgccgatattgtacaaggtcaaagagaacaggcaacatgtgctgtcg…
V L - L K L I M L M I H V V H F T K Y G L N P P K I K Q F H F S A D I V Q G Q R E Q A T C A V …

sDscamβ1-V1
atttcgtgaaacttatgtaatataatatcatcgtcgagaatggaatggcgtttagtatttgttctcacgtttccatttatgcatcaacaaataatgaattcgagccgaaaattcagccattctattttcctgaacgagtta…
I S - N L C N I I S S S R M E W R L V F V L T F P F Y A S T N N E F E P K I Q P F Y F P E R V …

sDscamβ1-V2
tattcttagaatattaacctagaaaagatgaattttcatcagaatatcagaggaataatcgcttggtttactgtgatttatatagttactgcaattcattctgaaagcggaattccgaatttacaaccgtttcattttcctg…
Y S - N I N L E K M N F H Q N I R G I I A W F T V I Y I V T A I H S E S G I P N L Q P F H F P …

sDscamβ1-V3
tttatataattaattgataccagtcttgcatactatacgaataattctagcattttctcggtgttcaatattgttttatgttgttcaatatacaataagagagtaatgatgatgtgtgtttgtttaacgatgttactgttca…
F I - L I D T S L A Y Y T N N S S I F S V F N I V L C C S I Y N K R V M M M C V C L T M L L F …

sDscamβ1-V4
gaaaattaaactagaaaaatgctgctgctcttctggataatttgcttcagtggatcgtttgtaattggagaaatccgtcgaagcggtacaacattaaaaattcagccatttttatttccctccaaacctatcagcgggtcaaa…
E N - T R K M L L L F W I I C F S G S F V I G E I R R S G T T L K I Q P F Y F P P N L S A G Q …

sDscamβ1-V5
aagaagtgagtagaaaggagccatcacacataccgttgtttgccactgactgcattgagtagcaacacaagaattgaggctaagtgtgtagaaatgaggctagaattactgattagtctcaatgcctggattctgagtatcg…
K K - V E R S H H T Y R C L P L T A L S S N T R I E A K C V E M R L E L L I S L N A W I L S I …

sDscamβ1-V6
tgtgtttagagtttacattcgaaatgattaatagtctgtttgtgatgatgtttcgatatggttcaccaccgtttcttccgacgcaccggataacaataatattccgaaaatacagcctttcaactttccgccaaatttgt…
C V - S F T F E M I N S L F V M M F S I W F T T V S S D A P D N N N I P K I Q P F N F P P N L …

sDscamβ1-V7
tttgagtagtatttgaaagttgaaattgggtacaacatattgagtagatatagaataatgatcaacaatcagaaaaatatttctgcagttctttatttgattatcttgaactacatcatggaagctttggcagcagaatctc…
F E - Y L K V E I G Y N I L S R Y R I M I N N Q K N I S A V L Y L I I L N Y I M E A L A A E S …

sDscamβ1-V8
gatatataaataaatatgcacttgagttgttcgttcagatctcagtgcgtgtttttgatactagtgacgacacaattaattcttctgacagcaatgaatgatgatattaaaattcaacctttttatgcttcctcataaaacat…
D I - I N M H L S C S F R S Q C V F L I L V T T Q L I L L T A M N D D I K I Q P F M L P H K T …

sDscamβ1-V9
ttcacataaacgtttaaaagtgcgtcttttataagaatgaataggcatttgatcttcgacttctatctttgtctactgactgttgttagaggcgatctgcttacaattcatcctcttattttccatctaaaataagcgaag…
F T - T F K S A S F I R M N R H L I F D F Y L C L L T V V R G D L L T I H P L I F P S K I S E …

sDscamβ1-V10
ttattttaagcattcgctattgcaattaaaacaaacaaaagatcaataatgttatcaaaaactgcagtaaaatatgtttgctttatctctatgtatcgcatcgttataggggtggtacctatcattcaaccattcacattac…
L F - A F A I A I K T N K R S I M L S K T A V K Y V C F I S M Y R I V I G V V P I I Q P F T L …

sDscamβ1-V11
gctgagtgatttggggttgtctttatgctatcgccatcttccaaatgcagtagtgattatattcacgtgtgtttaagacatttaccggtacatacatacataaatatgcaggtgtgttattcgttcagatctcgatacatat…
A E - F G V V F M L S P S S K C S S D Y I H V C L R H L P V H T Y I N M Q V C Y S F R S R Y I …

sDscamβ1-V12
atcacataaaacacacgaaagatgcttaaaagtgcttattttacgagaatgaataggcatttgatcttcggcttgcatctttatctactgggcattgtcagaggtgatttgctcacaattcatccgcttattttcccatcta…
I T - N T R K M L K S A Y F T R M N R H L I F G L H L Y L L G I V R G D L L T I H P L I F P S …

sDscamβ2-V1
cacatatgaatcgttattttaaaaactgtaataatgacattagcgaagagtgtaataattattgcattactcagtggactttcgtctgtctcaagcgaacttacggtacggaatttacatttccaaatgctgtatttataa…
H I - I V I L K T V I M T L A K S V I I I A L L S G L S S V S S E L T V R N F T F P N A V F I …

sDscamβ2-V2
tgtatttaaaactatggagatcgtttatgtcgtaaatctaaggatatagatcgtacgcaagggataaaggtagcgattcttgataaatattcgaaaatgactatttaaaccgcttcttgtggattttagttatggaattaa…
C I - N Y G D R L C R K S K D I D R T Q G I K V A I L D K Y S K M T I L N R F L W I L V M E L …

sDscamβ2-V3
tcgttttagaagaataaccagctatctacaatgaatttattcagtctgtatttaaatacatttttatcacttggaatcatgtctattgttatatcagaggaactgcacatccctgcctttaattttcgagataatgttatgg…
S F - K N N Q L S T M N L F S L Y L N T F L S L G I M S I V I S E E L H I P A F N F R D N V M …

sDscamβ2-V4
tatcaatagaacattataaaaataatgaaaataattttgaggattgtatcttcgatcactttagtcgtgcttgctgcaactgaggaaggaatacctaggcttcaacccatatttttcctgaatttgtatcagtgggagaaa…
Y Q - N I I K I M K I I L R I V S S I T L V V L A A T E E G I P R L Q P I F F P E F V S V G E …

sDscamβ2-V5
gttgactgaacattacttaattcggtttatgtt………atttgagtagaaagagattttcacaacaaaaatatagtgttcattctgtaaaggacgtttcatatggtcatccgcagaacgttattaaaataatgaaaacaa…
V D - T L L N S V Y V ………I L S R K R F S Q Q K Y S V H S V K D V S Y G H P Q N V I K I M K T…

sDscamβ2-V6
cataattgattttattcaagaatattttttaatgaagatgactttactgttaagttttgtgtttactcaattcttggcagtgttcggaggtgaattgaatgtatcaccatttgttttccgagaaaatgtcatggtaggcgaaa…
H N - F Y S R I F L M K M T L L L S F V F T Q F L A V F G G E L N V S P F V F R E N V M V G E …

sDscamβ2-V7
ctgacctaatttcaaagaataatcgagaaaatgtggactcttaaagttgtattgagtgttgtattaccgacttggatatactgtgaagaaggtggtccaaagattcagccgttttattttcccgaaaatgtaaatgtcggac…
L T - F Q R I I E K M W T L K V V L S V V L P T W I Y C E E G G P K I Q P F Y F P E N V N V G …

sDscamβ2-V8
accaattaaacgtcactgtgtctctgtgatgatctcagctatttagtgagattaatacaaatcatgtatacttgctggataatagcattgttgttaatgctgatactagaagaaacgaactgcaactcagatgctcctaaaa…
T N - T S L C L C D D L S Y L V R L I Q I M Y T C W I I A L L L M L I L E E T N C N S D A P K …

sDscamβ3-V1
aaaaagtgatttaatgatcaccatttaaagaaaatatgttgatattaatattgttaatatttacttttgcaattgcagatcaagattctttaaaaatccagccattcaattttccttctccttctgccataaacgagcgtg…
K K - F N D H H F K E N M L I L I L L I F T F A I A D Q D S L K I Q P F N F P S P S A I N E R …

sDscamβ3-V2
aatatttaatcatcgataagaaagatgtacacgatatccaagcaaagaagtattgcaataataatcattgtgaactggatagttatacttgcaaacgagaggcttgaagttcaacctttcagttttcctgctataacatctg…
N I - S S I R K M Y T I S K Q R S I A I I I I V N W I V I L A N E R L E V Q P F S F P A I T S …

sDscamβ3-V3
tttgattgatgcagtatggccagactgaacttattgttgatattgaatatcgtagtcttctgtagtggaaaagatgatttgaaaattcagccattcaattttccaaaacaaccaattgttgggcaaagagtttcggtaatgt…
F D - C S M A R L N L L L I L N I V V F C S G K D D L K I Q P F N F P K Q P I V G Q R V S V M …

sDscamβ3-V4
agaatttgaaacaaattaaaatacaatttgttgaaaagtgtgtgtacagtaatagcgacgatgagcagattgttattcattttcatgctgcatcatttaatggaagtcaagtcacaagaattacctaaaatacagccattta…
R I - N K L K Y N L L K S V C T V I A T M S R L L F I F M L H H L M E V K S Q E L P K I Q P F …

sDscamβ3-V5
tccgtataaagatgcaaaatctgcatattaattgaagatatatatttacatataacaaacatgaaaatggcgttactactccttgtgatctcttttgtgttgatacttaacagactagtacaaagcttgtcacaagaattgc…
S V - R C K I C I L I E D I Y L H I T N M K M A L L L L V I S F V L I L N R L V Q S L S Q E L …

sDscamβ3-V6
gaaaactagtcattatatttagtttccaagtgtataaatattgtcgtgactattttcaagatgaagaattcgatgattgtatcagccatgttactgttatcttgtttagaagagattatatgccaaaatttacctaaactgc…
E N - S L Y L V S K C I N I V V T I F K M K N S M I V S A M L L L S C L E E I I C Q N L P K L …

Fig. S1 (continue)

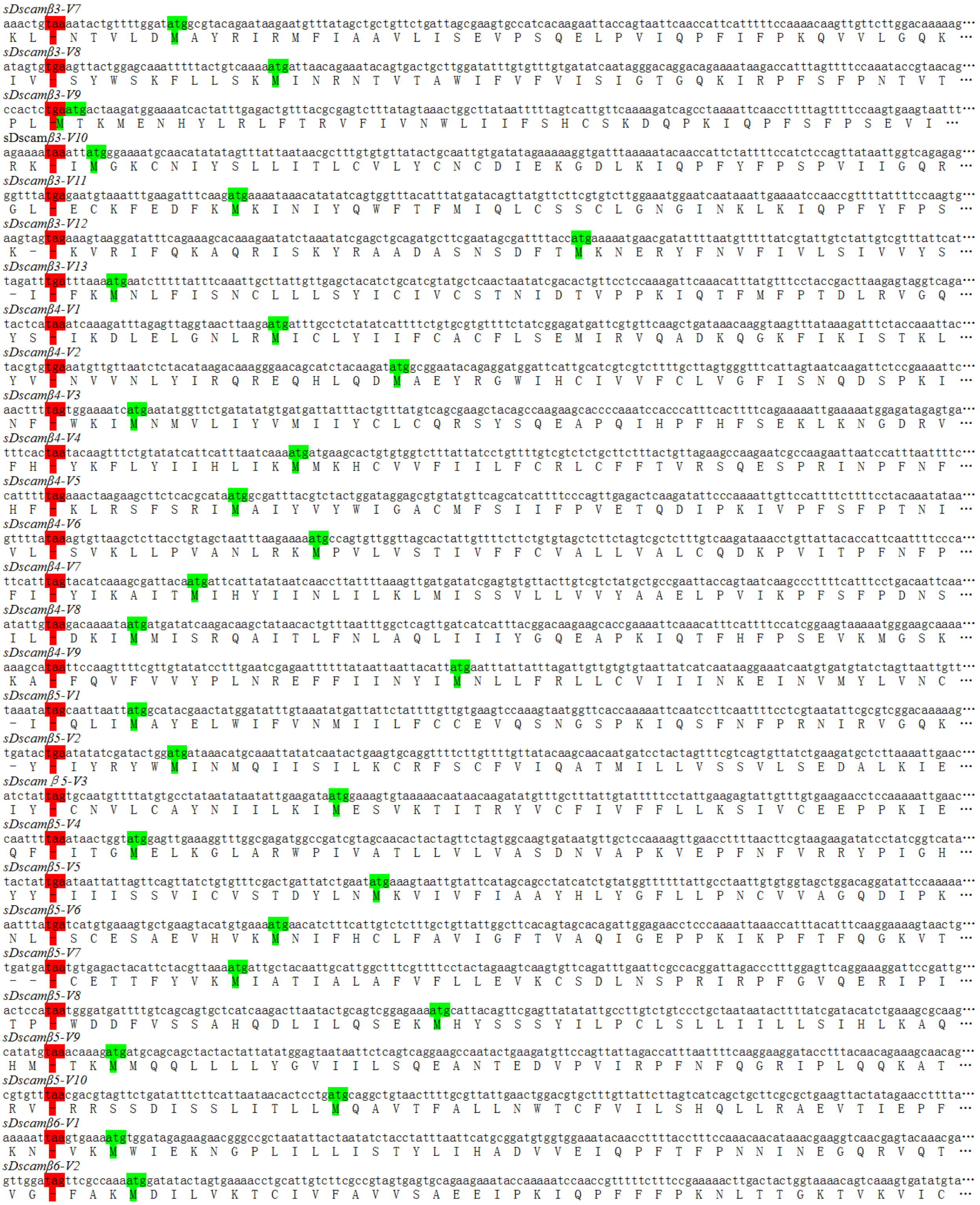

Fig. S1 (continue)

Supplementary Fig. 1: The feature of 5' variable cassettes of *M. martensii sDscam*. A stop codon (shaded in red) is generally located in frame immediately upstream from the ATG initiation codon (shaded in green) in each variable cluster. Almost all transcripts were confirmed by RNA-seq and RT-PCR. The putative translation start codon was determined by inspecting the translated signal peptide sequences.

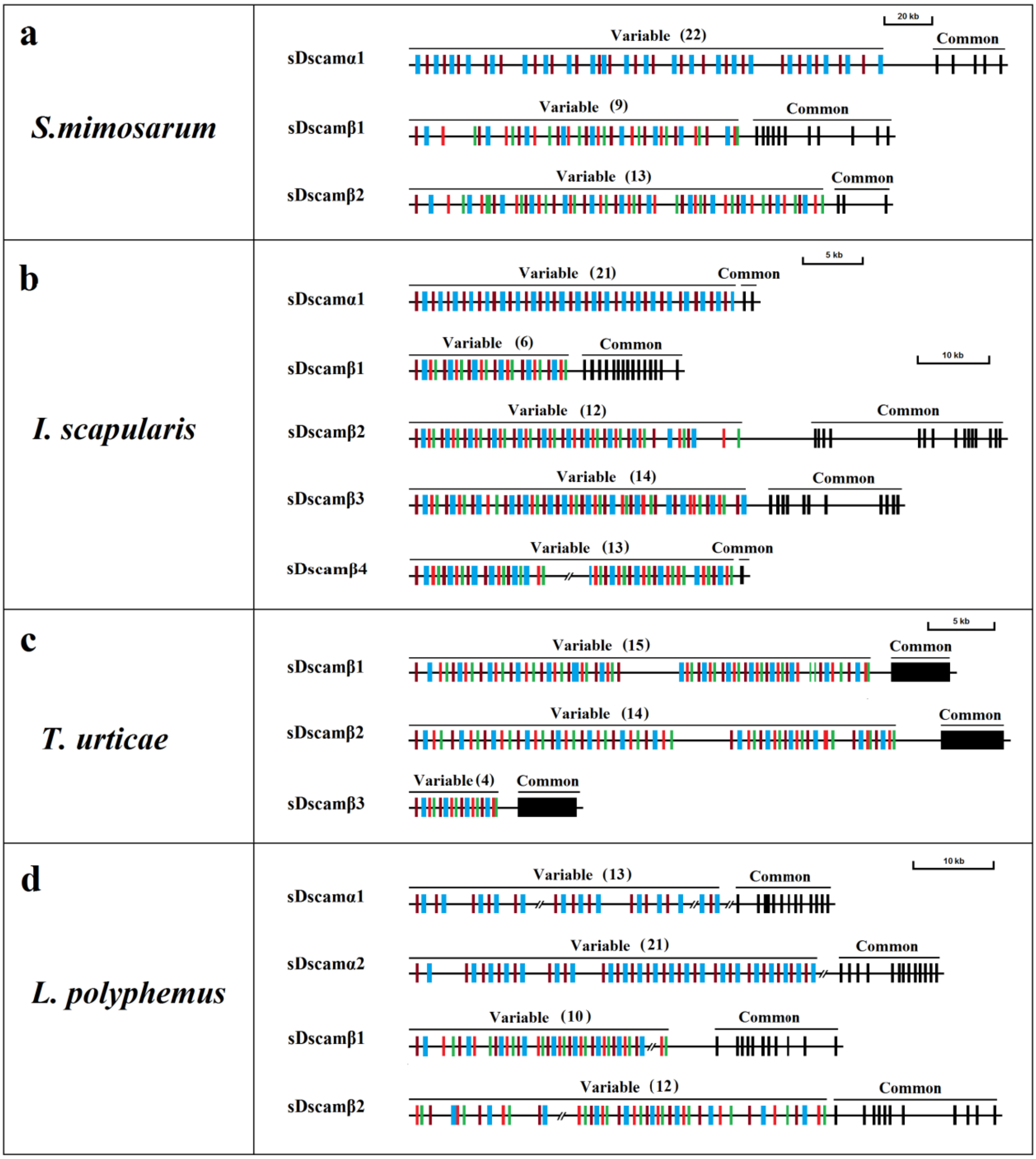

Supplementary Fig. 2: 5' clustered organization of *sDscam* is conserved in Chelicerata (Arthropoda). Organization of *sDscamα* and *Dscamβ* genes was shown in *Stegodyphus mimosarum* (a), *Ixodes scapularis* (b), *Tetranychus urticae* (c), and *Limulus polyphemus* (d). Symbols used are the same as in Figure 1. The exons are not drawn to scale. These numbers of tandem cassette (shown in parentheses) are likely to be modified when more accurate data become available, but this does not affect the general diagram. Ig, immunoglobulin domains;

FNIII, fibronectin III domains. *sDscamα* and *Dscamβ* genes are composed of multiple tandemly arrayed regions (indicated by the color boxes) and common region exons (indicated by the black boxes). *sDscamα* tandem cassettes are generally composed of two exons (indicated by the color boxes). *sDscamα* variable cassette encodes the N-terminal Ig1 (blue), which correspond to variable Ig7 domain of *Drosophila* Dscam1. *Dscamβ* tandem cassettes generally contain four exons. The variable cassette encodes the N-terminal Ig1+2 domains (colored), which correspond to variable Ig7+8 domains of *Drosophila* Dscam1. The exons and intergenic regions are drawn approximately to scale.

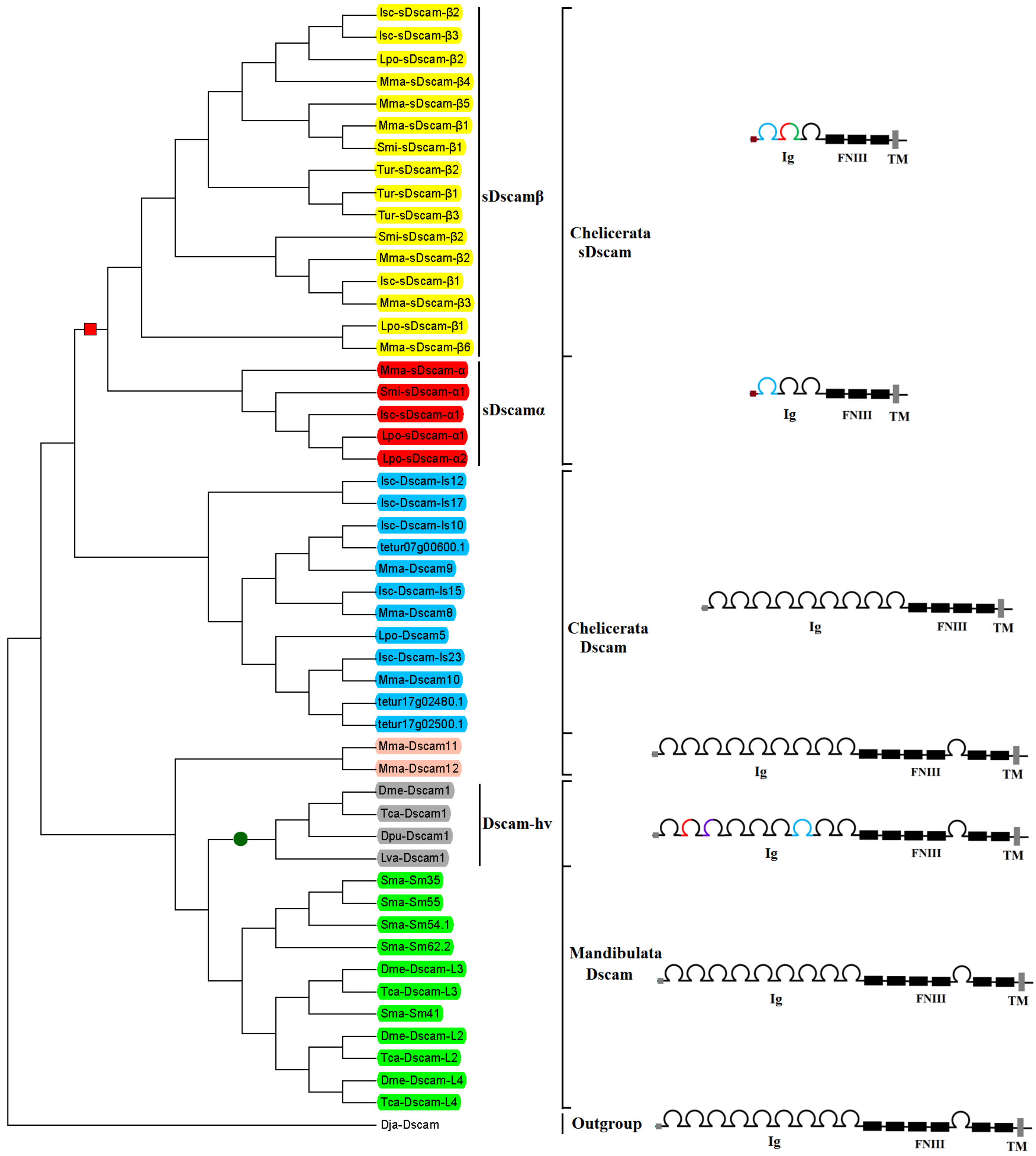

Supplementary Fig. 3: The evolutionary relationships and protein structures of arthropoda Dscams. The tree is based on amino acid sequence alignment of Dscam encompassing the seventh Ig domains to end (sDscam regions encompassing the first Ig domains to end), and is rooted using the sequence of the *D. japonica* (Dja) *Dscam1* (Ab249988). *M. martensii* (Mma) *sDscamα* (KT932407) and *sDscamβ1–6* (KT932408; KT932409; KT932410; KT932411; KT932412; KT932413); *L. polyphemus* (Lpo) *sDscamα1–2* (KT932388; KT932389) and *sDscamβ1–2* (KT932390; KT932391) are included. Other *sDscamα* and *sDscamβ* genes in *S.*

mimosarum (Smi), *I. scapularis* (Isc), and *T. urticae* (Tur) refer to Supplementary Table 1. *L. polyphemus* (Lpo) *Dscam5* (KT932392), *M. martensii* (Mma) *Dscam8–12* (KT932414–KT932406, KU378204–KU378205), *L. vannamei* (Lva) *Dscam1* (GQ154653), *D. melanogaster* (Dme) *Dscam1–4* (CG17800; CG42256; CG31190; CG42330), *T. castaneum* (Tca) *Dscam1–4* (NP_001107841.1; XP_967655.2; XM_963226; XM_967798), and *D. pulex Dscam1* (EU307884) are included. Other canonical *Dscam* sequences in *S. maritima*, *I. scapularis*, and *T. urticae* refer to recent references[1,2]. The emergence of *sDscam* is indicated by red squares, and the birth of *Dscam1* is indicated by solid circles.

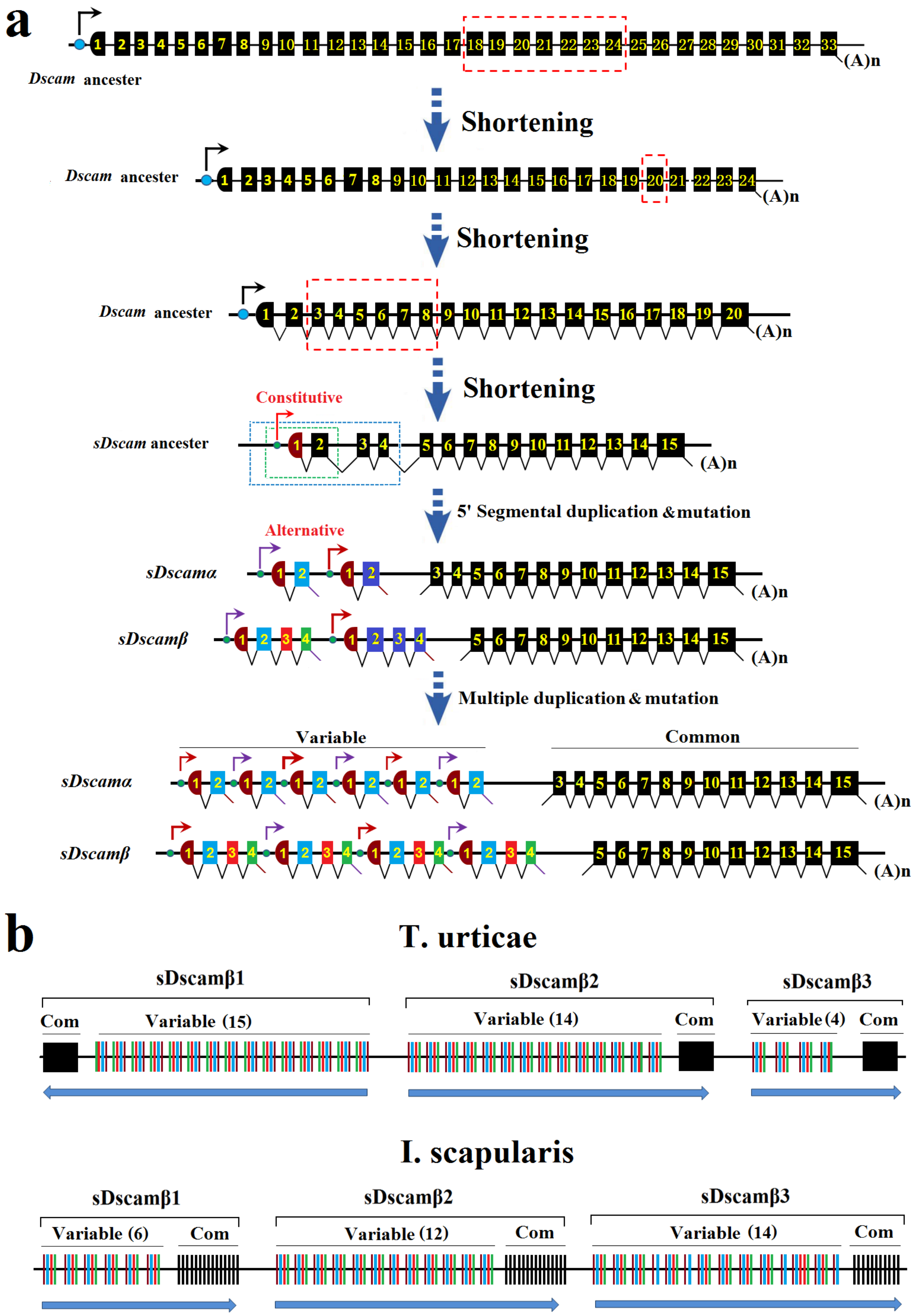

a
Dscam ancester
(A)n
Shortening
Dscam ancester
(A)n
Shortening
Dscam ancester
(A)n
Shortening
Constitutive
sDscam ancester
(A)n
5' Segmental duplication &mutation
Alternative
sDscamα
(A)n
sDscamβ
(A)n
Multiple duplication & mutation
Variable
Common
sDscamα
(A)n
sDscamβ
(A)n
b
T. urticae
sDscamβ1
Com
Variable (15)
sDscamβ2
Variable (14)
Com
sDscamβ3
Variable (4)
Com
I. scapularis
sDscamβ1
Variable (6)
Com
sDscamβ2
Variable (12)
Com
sDscamβ3
Variable (14)
Com

Supplementary Fig. 4: Origin and lineage-specific expansion of 5' clustered *sDscam*. (**a**) The model for the origin and expansion of 5' clustered *sDscam*. Symbols used are the same as in Figure 1. Circles represent promoter and arrows indicate transcription direction. First, the ancestral *Dscam* gene underwent sequential shortening of the Ig and FNIII domains of the canonical *Dscam* (marked by the red dashed box). Eventually, a shortened *Dscam* evolved in the ancestral gene. This *sDscam* ancestor was followed later by 5' segmental duplication to create 2 or more tandemly arrayed cassettes. The duplication unit may have included both exons 1–2 encoding a single Ig domain or exons 1–4 encoding 2 Ig domains and their promoters (green or blue dashed boxes). Mutations in exons and/or regulatory sequences optimized this gene regulatory system. Thus, various isoforms with diverse Ig1 (sDscamα) and Ig1–2 (sDscamβ) were generated by combining alternative promoters with alternative splicing. (**b**) Tandem gene clustered organization of Chelicerata *sDscams.* The *T. urticae Dscam* organization is based on the sequences (CAEY01001357), and that of *I. scapularis* partly refers to recent reference[1]. The introns are not drawn to scale. Arrow indicates the transcription direction. Abbreviation: Com, common.

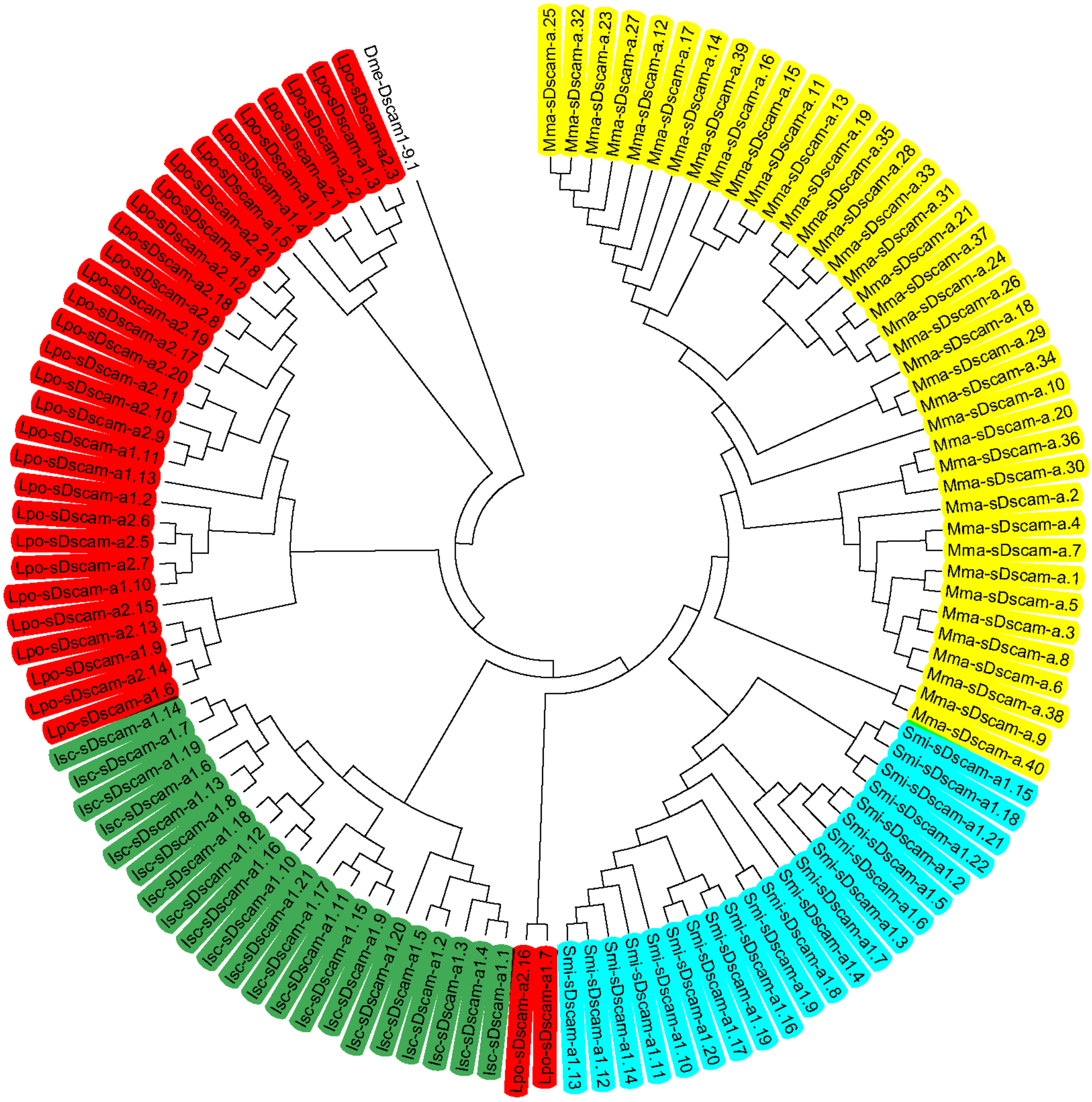

Supplementary Fig. 5: The evolutionary analysis of the duplicated cassettes encoding Ig1 domain of *sDscamα* in Chelicerata. The tree is rooted using *D. melanogaster Dscam1* duplicated exon 9.1 encoding Ig7 domain. *M. martensii* (Mma) taxon labels are yellow, *S. mimosarum* (Smi) blue, and *I. scapularis* (Isc) green, and *L. polyphemus* (Lpo) red.

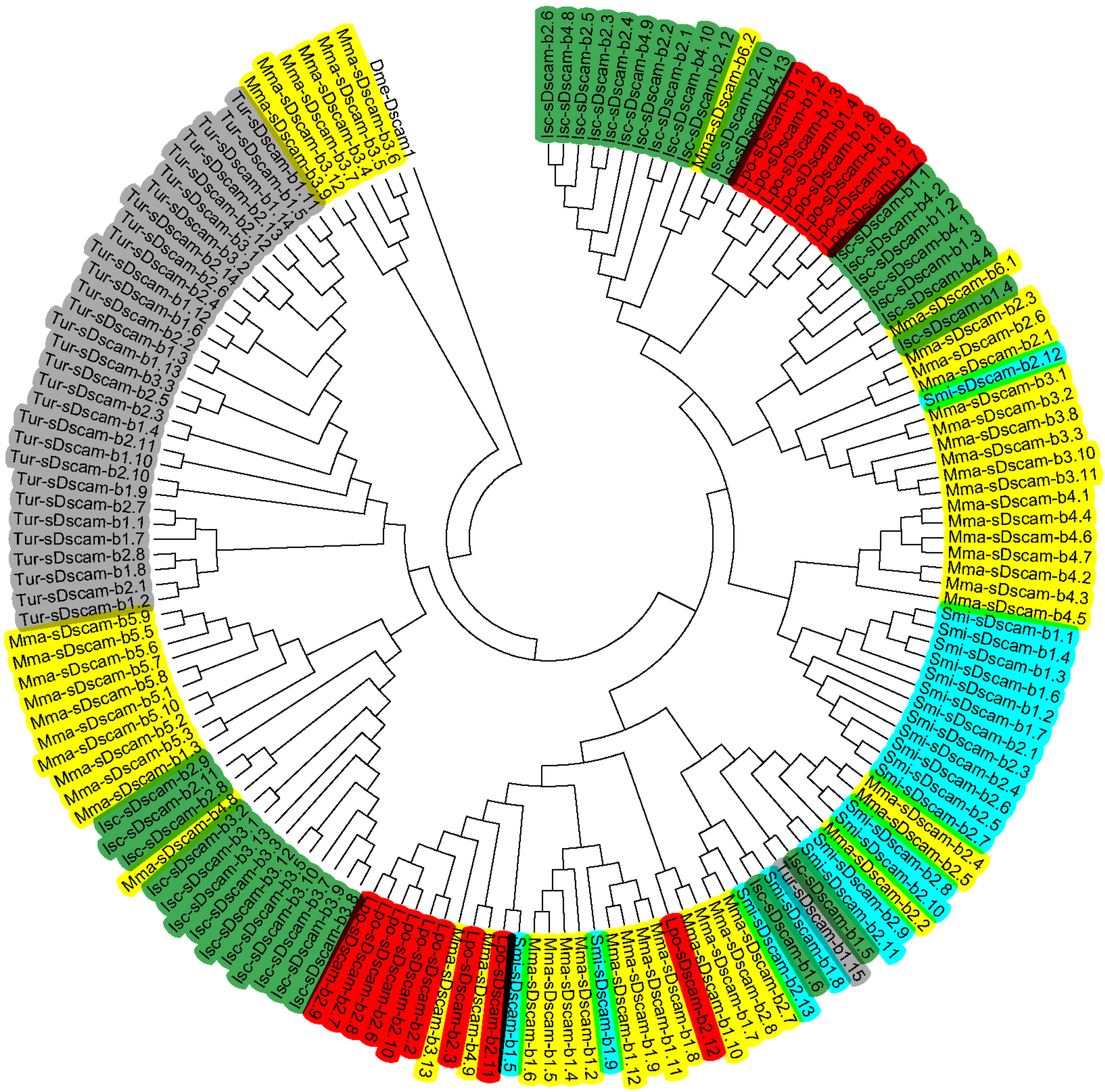

Supplementary Fig. 6: The evolutionary analysis of the duplicated cassettes encoding Ig1+2 domains of *sDscamβ* in Chelicerata. The tree is rooted using *D. melanogaster Dscam1* exon 9.1, 10 and 11 encoding Ig7+8 domains. *M. martensii* (Mma) taxon labels are yellow, *S. mimosarum* (Smi) blue, *I. scapularis* (Isc) green, and *T. urticae* (Tur) grey, and *L. polyphemus* (Lpo) red.

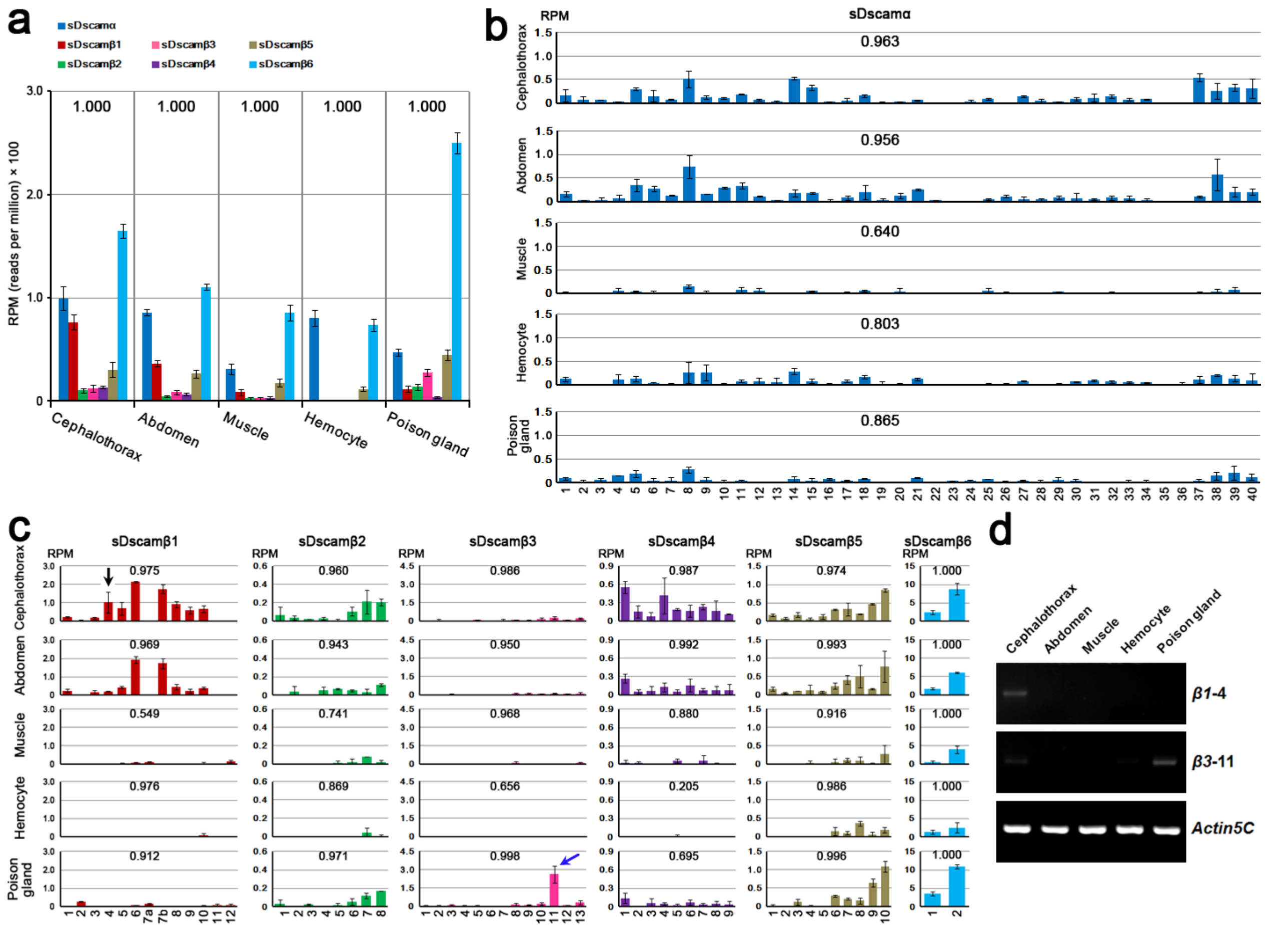

Supplementary Fig. 7: Expression analysis of 5' variable exons of *M. martensii sDscam*. (**a**) Relative expression levels of *sDscamα* and *sDscamβ1–6* transcripts in different tissues. The expression level for each transcript is shown as reads per million (RPM) of its corresponding constitutive exons based on the 50-nt fragmented RNA-seq datasets. Data are expressed as a percentage of the mean ± the standard deviation (SD) from two independent experiments. (**b**) The relative inclusion frequency of the *sDscamα* variable exon in different tissues. Alternative exon 2 was selected to calculate the expression level. (**c**) The relative frequency of the variable exon clusters of *sDscamβ1–6*. Variable cassette 4 of *sDscamβ1* was abundantly expressed in the cephalothorax (shown as the black arrow), but was barely detectable in the abdomen. *sDscamβ3* variable cassette 11 was abundantly expressed in the poison gland (shown as the blue arrow), but was barely detectable in other tissues. The correlation coefficient between the 25- and 50-nt mapping results was shown. (**d**) RT-PCR analysis of the variable exon 2.4 of *sDscamβ1* (*β1*-4) and the variable exon 2.11 of *sDscamβ3* (*β3*-11). Total RNA from different tissues was analyzed

by RT-PCR followed by electrophoresis. *Actin5C* was used as the internal control gene. These results indicate that these tissue-specific expression patterns are largely reproducible.

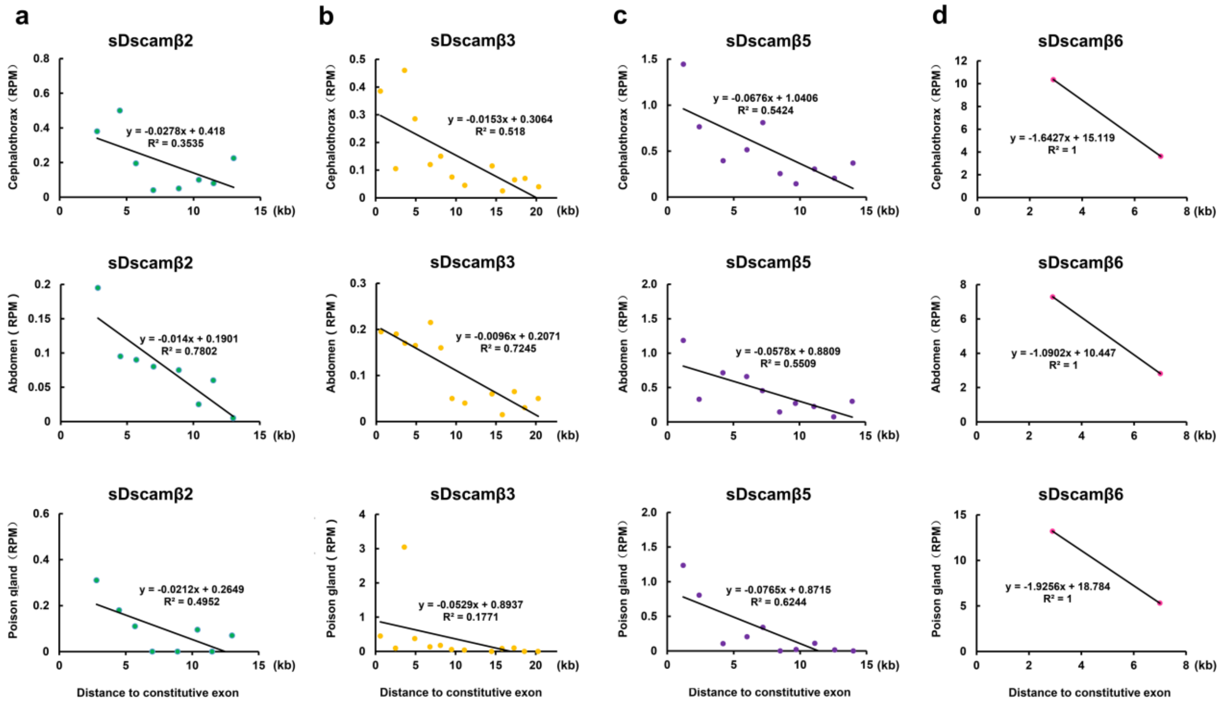

Supplementary Fig. 8: The inclusion frequency of a variable exon largely correlated with the proximity to the first constitutive exon. Since these *sDscams* were lowly expressed in muscles and hemocytes (Fig. 3a), we have not analyzed their correlation. Alternative exon 2 encoding Ig1 domain was selected to calculate the expression level. The expression level of alternative exon 2 is represented by RPM (reads per million). The relative positions to the start nucleotide of common region exon 1 are shown at the beginning and end of each sequence.

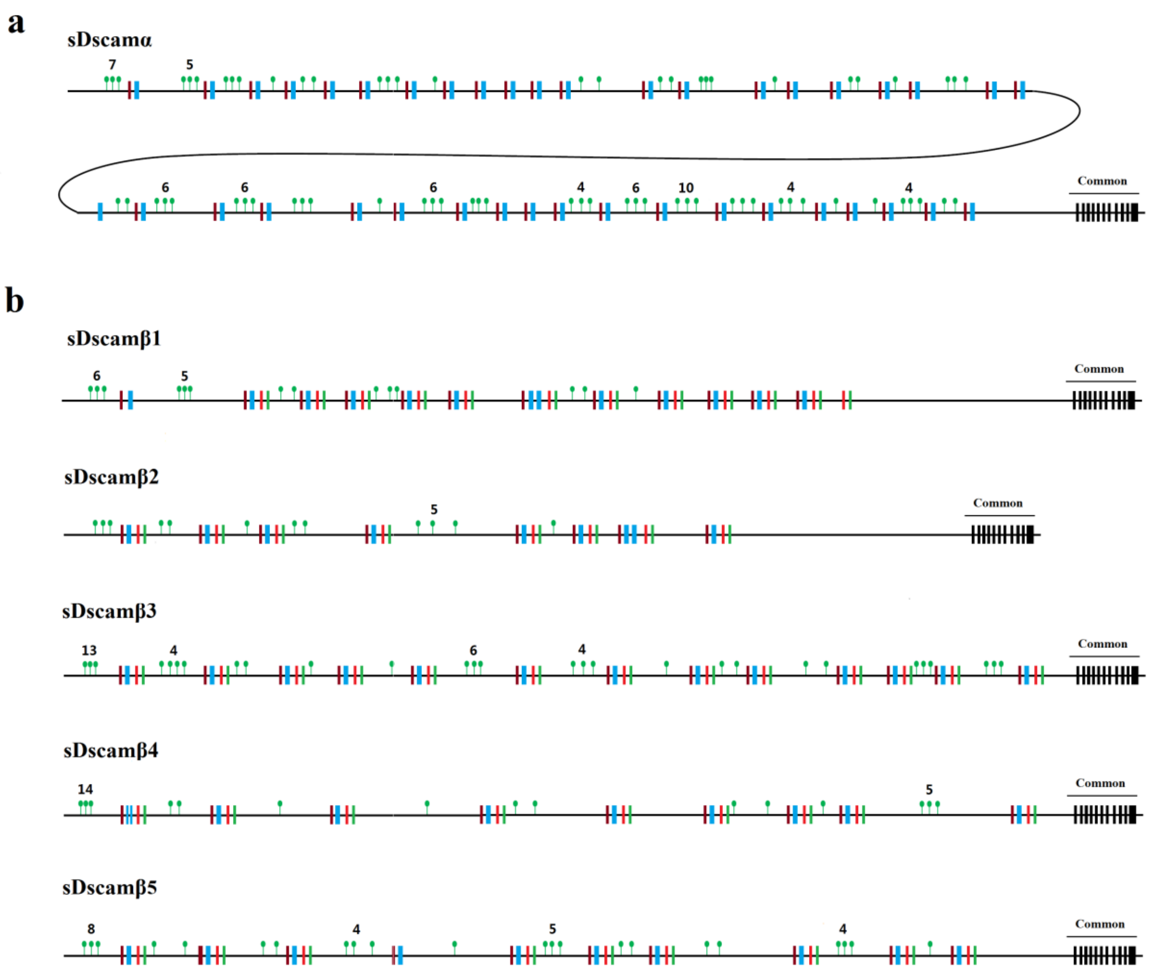

Supplementary Fig. 9: Potential promoter elements were predicted to be located upstream of the 5' end of each variable region of *sDscamα* (a) and *sDscamβ1–5* (b). The introns are not drawn to scale. Symbols used are the same as in Figure 5. The promoter distribution was predicted using the program (http://www.fruitfly.org/seq_tools/promoter.html). Potential promoter elements (PPE) are shown as green circles. In the case that there are more than three PPEs, only three are indicated with PPE number above.

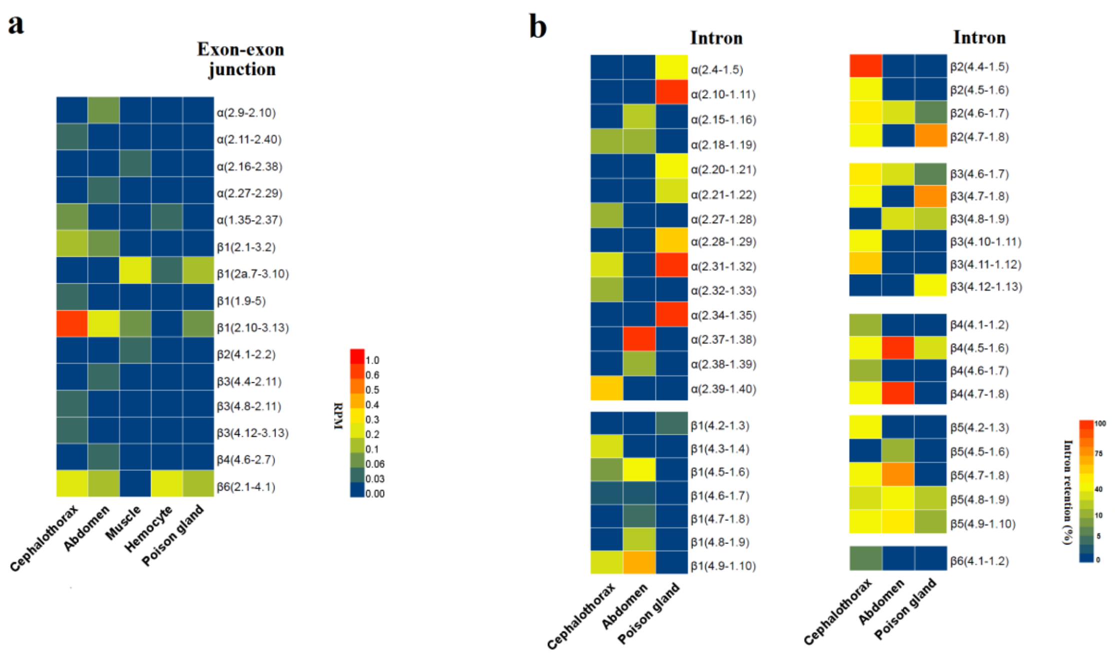

Supplementary Fig. 10: The expression of the non-canonical splicing isoforms of *sDscam*. (a) The expression of the non-canonical splicing isoforms in different tissues. Based on the exon-exon junction reads from RNA-seq data, we calculated the non-canonical splicing isoforms in different tissues. These results indicate that these splice isoforms are expressed in a tissue-specific manner. (**b)** Relative retention levels of within-cassette introns at 5' variable region in different tissues. Those without intron retention or undetectable expression are not shown. The expression levels of the within-cassette introns are shown, with red representing preferential selection and blue diminished representation.

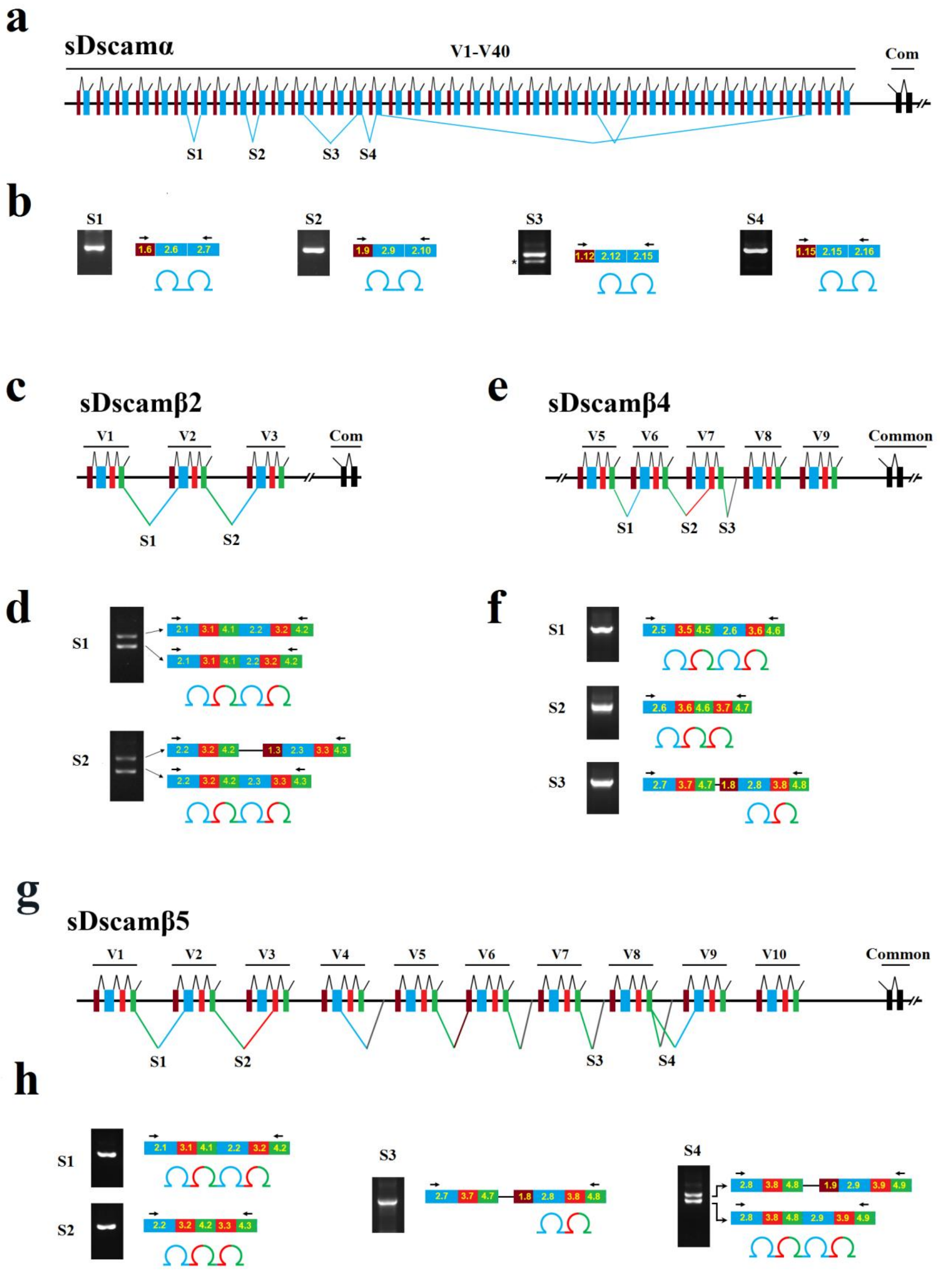

Supplementary Fig. 11: Highly complex combination of 5' variable exons in *M. martensii* *sDscam*. (**a**) Schematic diagram for splicing patterns of 5' variable exons in *M. martensii*

sDscamα. Symbols used are the same as in Figure 1. Splice isoforms within a single tandem cassette are indicated by black line above the gene structure diagram, while splice isoforms from different tandem cassettes are represented with colored line below, which was based on RNA-seq analysis and/or RT-PCR validation. (**b**) Alternative splicing junctions from different cassettes were validated using RT-PCR. Due to the low expression of *sDscam* variable exons, nested PCR is necessary to amplify products; only the primers for the second PCR are depicted, and same in panels below. The RT-PCR products were confirmed by cloning and sequencing. (**c**) Splicing patterns of 5' variable exons in *sDscamβ2*. (**d**) RT-PCR was used to detect *sDscamβ2* splice isoforms. (**e**) Splicing patterns of 5' variable exons in *sDscamβ4*. (**f**) Alternative splicing junctions from different cassettes were validated using RT-PCR with specific primers. (**g**) Splicing patterns of 5' variable exons in *sDscamβ5*. (**h**) RT-PCR was used to detect *sDscamβ5* splice isoforms.

Supplementary Table 1: A list of *sDscam* homologues identified in Chelicerata species.

	Gene name	Gene ID
Mesobuthus martensii (Mma)	*sDscamα*	AYEL01088921.1, AYEL01007825.1, KT932416, AYEL01077398.1,AYEL01015940.1,AYEL01063089.1
	sDscamβ1	AYEL01047811.1
	sDscamβ2	AYEL01089995.1
	sDscamβ3	AYEL01065522.1
	sDscamβ4	AYEL01079930.1, AYEL01050888.1
	sDscamβ5	AYEL01041827,KT932417,AYEL01076766.1
	sDscamβ6	AYEL01076766.1,KT932415,AYEL01084239.1
Stegodyphus mimosarum (Smi)	*sDscamα1*	AZAQ01125727.1
	sDscamβ1	KK120761.1(AZAQ01106106)
	sDscamβ2	AZAQ01116366
Tetranychus urticae (Tur)	*sDscamβ1*	CAEY01001357
	sDscamβ2	CAEY01001357
	sDscamβ3	CAEY01001357
Ixodes scapularis (Isc)	*sDscamα1*	ABJB010730407
	sDscamβ1	DS650268
	sDscamβ2	DS650268
	sDscamβ3	DS650268
	sDscamβ4	DS645963.1
Limulus polyphemus (Lpo)	*sDscamα1*	AZTN01155445.1, KT932395, AZTN01155444.1, AZTN01155443.1,AZTN01155442.1,AZTN01155441.1, KT932396, KT932397, KT932398, KT932399, AZTN01192115.1,AZTN01229939,AZTN01196346
	sDscamα2	AZTN01075309.1, KT932400, AZTN01075308.1, KT932401,AZTN01075307.1,KT932402,AZTN01075306.1,KT932403,AZTN01075305.1,KT932404, AZTN01075304.1,AZTN01075303.1
	sDscamβ1	AZTN01152876.1,AZTN01168237.1
	sDscamβ2	AZTN01029097.1,AZTN01029096.1,AZTN01029095.1, KT932393,AZTN01029094.1,KT932394, AZTN01029093.1,AZTN01029092,AZTN01029090, AZTN01029089

Supplementary Table 2: Primers used for the RT-PCR and PCR analysis

Primer	Sequence	Primer	Sequence
Mma β1-5-1	CCATTCTATTTTCCTGAACGAG	Mma β1-3-1	TCCCTCCACTTTTGCCGAGTTCCAC
Mma β1-5-2	GTGGTACCTATCATTCAACCAT	Mma β1-3-2	TGAAACTGTTTTCATGAGACTTCGC
Mma β1-5-3	AACTTTCCACCCAATTTATCTG	Mma β1-3-3	TCAAACTCTGTCCAACCCCATTATC
Mma β1-5-4	TTTCCGCCAAATTTGTCTGTTG	Mma β1-3-4	AAAACCCGTTGTCTTCTCTAGTCAC
Mma β1-5-5	GGTGCTCATCCAGTCAATTTTC	Mma β1-3-5	TTATTTCTTTATTAAGCGCTTCTCC
Mma β1-5-6	ACTTTAATCGAAGGTGCTGATC	Mma β1-3-6	TTGTGCATAAATATCTTCCACTGTC
Mma β1-5-7	TTTCCGCCAAATTTGTCTGTTG	Mma β1-3-7	TCTTTTTATTCAACGATTTTCCAAC
Mma β1-5-8	ACTTTAATCGAAGGTGCTGATC	Mma β1-3-8	CTGTGCATTCGTATTCGCCAGCATC
Mma β1-5-9	ATTCAACCTTTTATGCTTCCTC	Mma β1-3-9	GTACTGTCACACGAATAACTTTTTG
Mma β1-5-10	TTATGTAGCGTGACTAAAGGAG	Mma β1-3-10	CAATATCGTTTTCGGCCTTACATAC
Mma β1-5-11	GCCACTGACTGCATTGAGTAGCAAC	Mma β1-3-11	GAGGTTGATCCCCCGTTGCCTCGCA
Mma β1-5-12	ATTGAGGCTAAGTGTGTAGAAATG	Mma β2-3-1	TTGATAATTCCGAAATTTCTAATTC
Mma β1-5-13	TATTTCCCTCCAAACCTATCAG	Mma β2-3-2	CTGCAGTTTTCTTCAACGAAGGAAC
Mma β1-5-14	CAAGGTTCATATCCTGTTAATTTCG	Mma β2-3-3	ACATATAATTTCCACTATCTTC
Mma β2-5-1	ACTACTTGCATCACTAAGCAAGGCG	Mma β2-3-4	GTACTTTTCCCACTCCATTGCTTGC
Mma β2-5-2	TTCTCAGATCTATCTACTATCG	Mma β2-3-5	CTTCATCATCTTTTGTTGCCTGTGG
Mma β2-5-3	GGTTCGAAACCTCTAAAATATAAATG	Mma β2-3-6	CAATTCCGTTAGAAGCTTGGCATTC
Mma β2-5-4	TTATCTATAGATATGCAAACAG	Mma β2-3-7	TCCTTAAAAGATTTAATGTGCC
Mma β2-5-5	CCTCTGAAATTTAAATGGACTAAAG	Mma β2-3-8	TCGCTACAGCGTTCAATTTTTCACC
Mma β2-5-6	AATGTTTCTGTTGATGTTAATG	Mma β2-3-9	CGCATTTATAATTACCGCTATCTTC
Mma β2-5-7	TGTACTACAGTCACGGAAGATGCAC	Mma β2-3-10	TTATTATTGAAATCGGTTTTCCAAC
Mma β2-5-8	GACAACGAGCATATTAAAGTGC	Mma β2-3-11	TGCATTCATACTGACCACTGTCTTC
Mma β2-5-9	ACAGGAGACATGCCTTTACATTTTC	Mma β3 -3-1	CTTTTGACCAAGTGATGGTCATTGG
Mma β2-5-10	GAGTCTAATGTAGAAATTGGAC	Mma β3-3-2	TTTTGCAAGTATATAATCCTTCATC
Mma β3-5-1	AAAGAAATTAAGAAAAATAGCAATG	Mma β3-3-3	CCGTTATTATAAGTGTTCCATTTTG
Mma β3-5-2	GGCGATATATCGACTATAGTGATCG	Mma β3-3-4	TGTAATCTGCTCTTTCGATTACCAG
Mma β3-5-3	GAAATTAATACAGATGAACGACTCC	Mma β3-3-5	CTGCAGATGTTCGTTGATGTTC
Mma β3-5-4	ACTGTACAGGTTAACGATAATG	Mma β3-3-6	TAACAGATTTTATAATCATTGTGCC
Mma β3-5-5	GAAATCATTAATAAGGGACATGTTC	Mma β3-3-7	TCGAGCCTTCTTTCATTGAAATGGT
Mma β3-5-6	TCCTACGGTGATTTATCAAATATCG	Mma β3-3-8	CTAAATCTGTAACTTTATCTAAAAC
Mma β3-5-7	GAGCTGCAAAAGGATGAGAAAATCG	Mma β3-3-9	TACCAGGATACTGTTTTTGCGTTTG
Mma β3-5-8	GCCGATATTTCGACTATCATCATAG	Mma β3-3-10	CATCGCACTTCAAAGTAGCACTATC
Mma β3-5-9	GAGCTGCAAAAGGATGAGAAAATCG	Mma β4-3-1:	TTTAACTTTCCTCTCGTCGGTTCC
Mma β3-5-10	GCCGATATTTCGACTATCATCATAG	Mma β4-3-2	CTTTAACAGTAACAGTGATGGCTTT
Mma β4-5-1	AAGGATCACAGTGATATTAGTGAAG	Mma β4-3-3	TGGCTATGCACTCATAAATCCCTTC
Mma β4-5-2	GTGCTCACACATTCAGAATTGT	Mma β4-3-4	TACTCAAAGTGATAGTCTTCTTCAG
Mma β4-5-3	ATGCCAGTGTTGGTTAGCACTATTG	Mma β4-3-5	CGATGCCGTTATTAGCGATACATTC

Mma β4-5-4	TTCTTCTGTGTAGCTCTTCTAG	Mma β4-3-6	GTATTATAACTGATATTATCCTTCT
Mma β4-5-5	ATGATTCATTATATAATCAACC	Mma β4-3-7	GAGGTATTCCATTATCTGCTTC
Mma β4-5-6	TTAAAGTTGATGATATCGAGTG	Mma β5-3-1	CGGCTTTGCTTAATTTAATATCTTC
Mma β5-5-1	GTAATATTCGCGTCGGACAAAAAG	Mma β5-3-2	GTGAAGGCGAAATTCCATTGTCTGC
Mma β5-5-2	CTTGACATTTGAATGGTTGAAAGAC	Mma β5-3-3	ATCCTTGATCTTTTTCATTTGCCTC
Mma β5-5-3	GAAGAGTTCCAATAGGCGAAACAAC	Mma β5-3-4	TAGCTTGTCCAACATTGTTGTTAGC
Mma β5-5-4	GTTCAGTTACATCTAAAATTAAAG	Mma β5-3-5	AACCAGCTATATCATCAGAAGC
Mma β5-5-5	CTTTGGAGTTCAGGAAAGGATTC	Mma β5-3-6	CCACAACATGCACTTGTTTCATTAG
Mma β5-5-6	CTGGAAATTCTTTAGAGTTTAAATGG	Mma β5-3-7	TTAGAAGCTTCGCATTGATAAG
Mma β5-5-7	CAAAAATAAAGCCATTTAGTTTC	Mma β5-3-8	CTGTAATACGTTTCTTAATGCTCGG
Mma β5-5-8	CAGGGAGAAAAAGCTTTAGCTAC	Mma β5-3-9	CAGCTTCGCATTCGTAAATGCC
Mma α-5-1	TAAACATCTCATACAGATAATTG	Mma α-3-1	TATTAAGAGGCGGTTCACCAAATAC
Mma α-5-2	TATTTCAAGATACAACCGATAG	Mma α-3-2	GTATCAAAGAAGCTGTGAAGCTATC
Mma α-5-3	CATACTCATTATTACAGTGTAAGTC	Mma α-3-3	GCAGCATTCTTAACTATGCAAGT
Mma α-5-4	CAAAATCTATTATTTCCTTATAAC	Mma α-3-4	TTAATAGTGGAGCAGTATAGAAATC
Mma α-5-5	AATCATTTGTTGCTGGAATTCAG	Mma α-3-5	GTTTCCAATATTTTTAAACTGAAC
Mma α-5-6	ATCATTTCATGTCTGTGTTATAG	Mma α-3-6	GTATTAATGAAGCTGTATACCTATC
Mma α-5-7	GTTAGTTTTATTATAATTATTAAAC	Mma α-3-7	CCTAACAATGCACGTATAATTCCC
Mma α-5-8	GTTTCTTGGTATAGAAGTTCAA	Mma α-3-8	ATGGAGCTGTATATCTATCCGAACC
Mmaβ6V1-5-1	GGGGTACCCAAAACTATTTGTACTTCAGATA	Mma α-3-9	AGTAACAATGCACGTATAATTTCC
Mmaβ6V1-5-2	GGGGTACCTGACCATAAGTTCTCTTCGCCTTC	Mmaβ6V1-3-1	CCGCTCGAGACACAAACACAACTGGTTTCAAAG
Mmaβ6V1-5-3	GGGGTACCATCGAGCCGAAAATTCTGCCACAG	Mmaβ6V1-3-6	CGGAATTCGAAGGCGAAGAGAACTTATGGTCA
Mmaβ6V1-5-4	GGGGTACCTGTATCACACGAATGTACGGTGAG	Mmaβ6V1-3-7	CGGAATTCCGGCTATCATCTCAACTAATTGCT
Mmaβ6V1-5-5	GGGGTACCAGTCTGGTAGAATATGGACGTCAG	Mmaβ6V1-3-8	TTCAACTTCATATTAGGCGTTGTCTCTCAAGAAAAAATCATTCTT
Mmaβ6V1-5-6	CGGAATTCTCAATTAATCGAAAATTATGAAAG	Mmaβ6V2-3-1	CCGCTCGAGGCTGGAACTGACGTCACACAACA
Mmaβ6V1-5-7	CGGAATTCAGTCTGGTAGAATATGGACGTCAG	Mmaβ6V2-3-8	CCAAGCTTAGACAGTTGTGCTACTCGGGTG
Mmaβ6V1-5-8	AACGCCTAATATGAAGTTGAAAGTTAGTGTGACTTGACATAAATG	Mmaβ6V2-3-9	TCGTGCAGGTGTAATATACGTATATCCTTCTTG
Mmaβ6V2-5-1	GGGGTACCAGAAAGAAAACCCATATCTTGTC	Mmaβ6V2-3-10	TCCAATCTATGAATAGACTAATTCTGTAGCGTAT
Mmaβ6V2-5-2	GGGGTACCACTTAGCAAAAGCTGTCGTCCGG	Mmaβ6V2-3-11	TCCATCCAATCTATAGACAGTTGTGCTACTCGGGTG
Mmaβ6V2-5-3	GGGGTACCGAGAGACACGAAGTACACATTATC	Mmaβ6-3-12	CCGCTCGAGACATCGCAAAAATTAATTACCTG
Mmaβ6V2-5-4	GGGGTACCATGTTGTTAAATCGAGCAAGAAGG	Mmaβ6V2-5-9	CGTATATTACACCTGCACGACTATTCTATTATC
Mmaβ6V2-5-5	GGGGTACCATTTCCCCTCTCCTTTTAAATCG	Mmaβ6V2-5-10	TAGTCTATTCATAGATTGGATGGAAGAAAAGTC
Mmaβ6V2-5-6	GGGGTACCTGTTATCGTTATATAAACGATAAT	Mmaβ6V2-5-11	TAGCACAACTGTCTATAGATTGGATGGAAGAAAAGTC
Mmaβ6V2-5-7	GGGGTACCTAGTAGTATCGCGATCGATAAAC	Mmaβ6-5-12	GGGGTACCTAGCCGTTAAATAATTAAATTACG
Mmaβ6V2-5-8	CCAAGCTTACTTCGCTTAGCTGTCAATC	DmeDs2-3-1	CCGCTCGAGCTCTTCGGCGAGAATTCACCACA
DmeDs2-5-1	GGGGTACCACGATGCATGGACATGGGGAGC	Mma actin1-3	CAAGATAGCGTGGGGAAGAG
Mma actin1-5	GTTACCAATTGGGACGATATGG		

Supplementary References

1. Brites, D., Brena, C., Ebert, D. & Du Pasquier, L. More than one way to produce protein diversity: duplication and limited alternative splicing of an adhesion molecule gene in basal arthropods. *Evolution* **67**, 2999-3011 (2013).
2. Palmer, W.J. & Jiggins, F.M. Comparative genomics reveals the origins and diversity of arthropod immune systems. *Mol. Biol. Evol.* **32**, 2111-2129 (2015).
